# Embedding Nonrigid Solutes in an Averaged Environment: A Case Study on Rhodopsins

**DOI:** 10.1021/acs.jctc.3c00285

**Published:** 2023-07-13

**Authors:** Niccolò Ricardi, Cristina E. González-Espinoza, Suliman Adam, Jonathan R. Church, Igor Schapiro, Tomasz Adam Wesołowski

**Affiliations:** † Department of Physical Chemistry, University of Geneva, 1205 Geneva, Switzerland; ‡ Fritz Haber Center for Molecular Dynamics, Hebrew University of Jerusalem Israel, 91904 Jerusalem, Israel

## Abstract

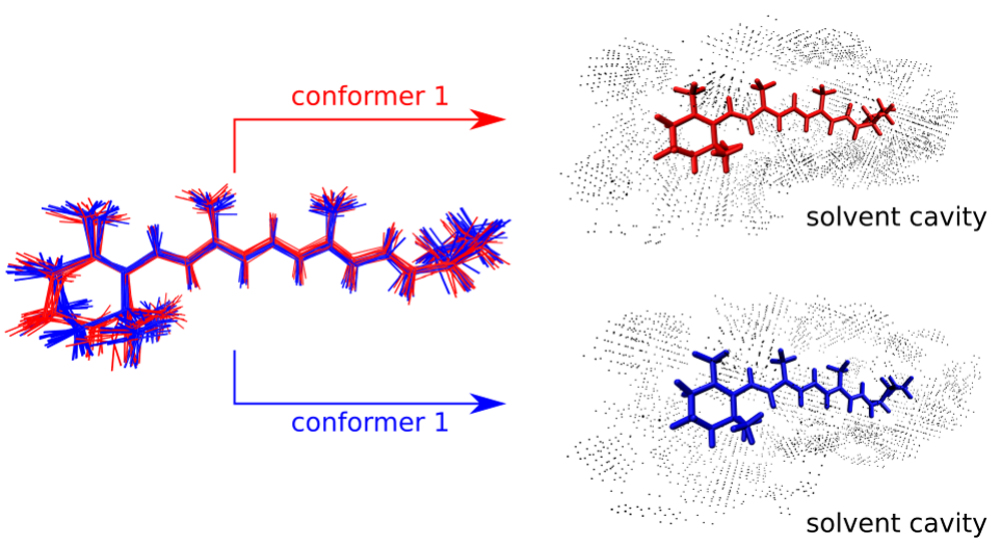

Many simulation methods concerning solvated molecules are based on the assumption that the solvated species and the solvent can be characterized by some representative structures of the solute and some embedding potential corresponding to this structure. While the averaging of the solvent configurations to obtain an embedding potential has been studied in great detail, this hinges on a single solute structure representation. This assumption is re-examined and generalized for conformationally flexible solutes and tested on 4 nonrigid systems. In this generalized approach, the solute is characterized by a set of representative structures and the corresponding embedding potentials. The representative structures are identified by means of subdividing the statistical ensemble, which in this work is generated by a constant-temperature molecular dynamics simulation. The embedding potential defined in the Frozen-Density Embedding Theory is used to characterize the average effect of the solvent in each subensemble. The numerical examples concern the vertical excitation energies of protonated retinal Schiff bases in protein environments. It is comprehensively shown that subensemble averaging leads to huge computational savings compared with explicit averaging of the excitation energies in the whole ensemble while introducing only minor errors in the case of the systems examined.

## 1 Introduction

Modeling molecules in the condensed phase requires the simulation of large systems. This has led to the development of a multitude of multiscale approaches. Despite the variety of these methods, most of them are based on the division of the system into two subsystems: the system of interest, from hereon denoted as A, and the environment, from hereon denoted as B. Another challenging aspect is the need to extend the calculation also on the time scale, to account for the thermal agitation of the system. Sampling of the statistical ensemble, by means of molecular dynamics or molecular Monte Carlo, can be used to obtain a set of geometries  (divided into  and ) which adequately sample the free energy surface. The ensemble of observables  can then be investigated, where each value is obtained as
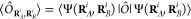
1

For the sake of simplicity, a single average value  may be considered, where *ens* denotes the set of samples of the statistical ensemble. Directly computing such a value would constitute a significant computational advantage. To this end, a *single solute structure representation* is commonly used, resulting in eq 2
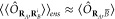
2where  generically denotes some descriptor of the average environment. This can be done in several ways, which are outlined below.

For instance, in continuum dielectric based solvent models,  is represented by means of the reaction field.^1−3^

At the atomistic representation of the solvent - i.e. if the ensemble of instantaneous structures  is available -  is the average electrostatic potential, such as the ones used in refs (4−17).

Recently, we have reported the statistical analysis of the fluctuations for solvated acetone,^18^ confirming the validity of the use of an average electrostatic potential.

Frozen-Density Embedding Theory (FDET)^19,20^ provides a straightforward interpretation of  that includes not only electrostatics but also a complete quantum-mechanical description of the whole system. In FDET,  represents two quantities: the statistically averaged electrostatic potential generated by the nuclei of the solvent ⟨*v*_*B*_⟩(**r**) and the statistically averaged electron density of the environment ⟨ρ_*B*_⟩(**r**). The FDET embedding potential in such a case reads^21^
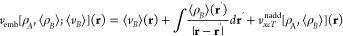
3where the first two terms account for the electrostatics (nuclear attraction and Coulomb repulsion), whereas the last one takes into account the non-classical effects of the environment on the embedded wave-function.

We used this approximation in modeling excitation energy shifts to determine the minimum number of structures necessary to correctly describe the ensemble.^18,22−24^

There may be several reasons to justify the use of a single solute structure representation approach to model subsystem A (**R**_*A*_ in eq 2). Semirigid molecules can simply be modeled by means of their gas-phase structures.^18,21−24^ In other cases, a structure is chosen which minimizes the free energy of the supersystem, with some multiscale method of choice.^5−9,11−17^

Albeit technically applicable to a set of local free energy minima,^25,26^ the latter approach is commonly applied to the global minimum only, relying on the assumption that the whole ensemble is well represented by a single structure within its average environment. Even when a set of structures is used, their observables are often individually compared to experimental values,^27^ rather than obtaining a single Boltzmann-weighted average value.

For nonrigid solutes, the analogue of eq 2, where also the solute coordinates are averaged, reads
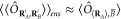
4

For a solvated molecule such that its structure oscillates slightly around a single conformation, it is reasonable to expect that the coordinates can somehow be averaged. Issues may nonetheless arise when the solute is nonrigid. A single average solute structure may be chemically unreasonable or significantly different from any of the conformers, with obvious risks for the quality of any observable pertaining to the structure. Direct averaging of a coordinate spanning two conformers can lead to a value similar to that expected for the transition state with the conformation change. For instance, let us imagine a system with a torsional angle Φ with two stable conformers characterized by the values Φ_1_ and Φ_2_ with very similar free energies *G*_1_ ≈ *G*_2_. The occurrence of the two conformers will be approximately equal, resulting in an average value , which is in turn similar to the angle, and consequently energy, of the transition state for the conformerism. Furthermore, the movement of the environment will generally be coupled to the movement of the solute, and failure to account for this may prevent the average environment from having a proper cavity for the solute due to the steric effect, in turn due to the neglect of the cavity adapting to the flexible solute. Moreover, if the instantaneous structures oscillate around an average one which has a higher symmetry, this approximation is expected to fail. For instance, quadrupolar solutes can assume an instantaneous dipole moment, which in turn can induce order in the environment.

One possible strategy to avoid such issues is based on dividing the statiscal ensemble into subensembles according to
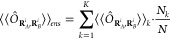
5where *N* and *N*_*k*_ are respectively the number of samples of the ensemble and those within the subensemble *k*. Then the approximation in eq 4 is applied to each subensemble, where it can be expected to be reasonably accurate, obtaining
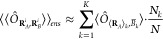
6where the expectation value inside the sum is given by
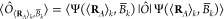
7For the specific case of FDET, eq 6 becomes
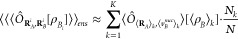
8

If the sampling represents the statistical equilibrium, the relative population in a subensemble is given by
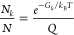
9where *G*_*k*_ is the free energy of conformer *k*, and *Q* is the system’s partition function. By introducing this in eq 6, one gets
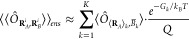
10

Eq 5 is exact regardless of how the division into *K* subensembles is performed. The approximation in eq 6 may be strongly dependent on the specifics of the division into subensembles. The focus of this article is to assess the accuracy of the approximation in eq 6 for the specific case of subensembles identified by the conformational changes of the system.

Various methods of clustering conformations were developed in different fields and can yield subensembles.^28−46^ Albeit direct clustering of the structures may be a valid and promising strategy, the results depend strongly on the selected subset of coordinates used for the clustering. This is why we instead started from the trajectory of each individual coordinate, which was analyzed, clustered, compared, and finally grouped to obtain the major conformation changes.

The averaging of both solute and environment is performed within each subensemble defined by the conformation change, from hereon called a “conformational basin”, according to eq 4. If *K* conformational basins are present, the final observable value is obtained as the weighted average of the values from the *K* average solute structures, weighted by the population *N*_*k*_ of each conformational basin (cf., eq 6).

For the sake of brevity, a simplified notation will be used from hereon: the quantum mechanical averaging to obtain the expectation value will be omitted, and observables obtained from a single calculation will be denoted as
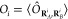
11for individual samples and
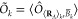
12when the average solute structure within its average environment, both for conformational basin *k*, is used. The symbols “⟨⟩” will then represent the averaging of values obtained from separate quantum mechanical calculations, and their subscript will denote the ensemble or subensemble over which the averaging takes place.

For our numerical testing, we will use the specific case of the distribution of the vertical excitation energy ε and the correponding oscillator strength *f*. Such distribution allows recovery of the inhomogeneous broadening of the spectrum, albeit not homogeneous broadening effects such as vibronic coupling and quantum decoherence. In such a case, the expectation values for the whole ensemble are obtained as
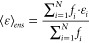
13
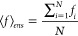
14This procedure is referred to as “ensemble averaging”. Conversely, the use of a few solute structures embedded in their average environment will be termed “basin-based geometric averaging”. Within a single conformational basin, where small oscillations around an average solute structure are assumed, the approximation of eq 4 is applied and excitation energies and oscillator strengths are simply calculated for the average solute structure for that basin in its average environment:
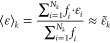
15
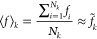
16

The averaging of the K basins {*k*} with populations *N*_*k*_ is then performed, and eq 6 for our chosen observables becomes
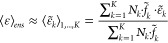
17
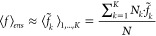
18

The principal area of applicability of basin-based geometric averaging is to obtain a single average value from subensembles based on clustering of geometric descriptors, which correspond to metastable structures, for instance, torsional angles, hydrogen bonding patterns, coordination number, etc. The proposed procedure may nonetheless be used in a more general context to validate the relation between a geometric descriptor and a quantum-mechanical observable. An example of such generalized application is the analysis of the correlation between bond length alternation (BLA) and excitation energy, discussed in subsection 3.3.

## 2 Methods

### 2.1 Detection of Basins and Structure Averaging

A detailed explanation or review of clustering algorithms lies outside the scope of this work. Most methods treat each structure as a point in an *n*-dimensional space, where *n* is the number of parameters considered, generally a set of coordinates, and then recognize clusters of points based on some distance metric. The results from trajectory clustering depend strongly on the clustering algorithm, on the distance metric used, and particularly on the selected atoms that it is applied to.^31^ This is why for this numerical test case we instead developed tools to aid the analysis of the individual trajectories of each internal coordinate. These are not to be considered a black-box automatic procedure to obtain average structures but a helping toolbox for the knowledgeable human operator.

The detection of these conformational changes was restricted to the solute only, but this may also be extended to the interaction of the solute with the solvent. For instance, different coordination numbers, distance, or partners may be detected throughout the trajectory. An example of such a situation is an arrangement of two hydrogen bond acceptors and one donor making it possible to form two distinct hydrogen bonds that alternate along the trajectory. These shall be considered and treated as two different basins rather than using an average environment with approximately half the charge density of each molecule.

The first step is alignment via rototranslation of the solute in each frame. This will be also necessary for the averaging of the solvent and is performed also for the averaging of rigid solutes. This rototranslation is performed via the Kabsch algorithm^47,48^ between any frame’s solute geometry  and the initial geometry . This minimizes the root-mean square deviation, according to
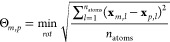
19where **x**_*m*,*l*_ and **x**_*p*,*l*_ represent the Cartesian coordinates (*x*, *y*, *z*) of the *l*-th atom, and *m* and *p* denote two general geometries. In this specific case, *m* refers to , and *p* refers to . The index *l* can span all atoms or a selected subset whose size is denoted by *n*_atoms_. Throughout this manuscript, unless specifically mentioned, all atoms have been considered.

The geometries of the solute were converted to internal coordinates, which we deemed more insightful than Cartesian ones. For instance, if one considers a bond that is bending without any stretching, averaging in Cartesian coordinates would lead to a shortened bond, while internal coordinates would leave the bond length unchanged and yield the average bond angle. The conversion to internal coordinates was performed with the Python module *chemcoord*.^49^ Nonetheless, very similar Cartesian coordinates may, in some cases, lead to discontinuity in the internal coordinates. One such case is that of dihedral angles close to ±180°. To solve this, the standard deviation of each dihedral angle is then evaluated in the −180°–180° and 0°–360° ranges. The angles for which the latter is smaller than the former are always analyzed in this range; finally, the average values are converted back to the conventional −180°–180° range. An initial screening of the internal coordinates can be done by analysis of the standard deviation: large values correspond to freely rotating groups; small values correspond to stable chemical groups, while medium values need further analyses. Jumps in the value of an internal coordinate can be detected calculating its average value every *x* frames, where *x* is a user chosen integer, and calculating the change between the average for a set of *x* values and that of the previous one. Large absolute values of this change correspond to jumps of value.

Clusters for each internal coordinate were finally obtained with a combination of two approaches:

- via clustering of the distribution, which was performed via x-means,^50^ in its *pyclustering*(51) implementation
- by first obtaining time domains via detection of *n*_*t*_ consecutive frames where the running average changes by more than a threshold τ, then grouping time domains whose average value is significantly close (user-defined threshold)

It is worth highlighting that the detection of jumps and time domains exploits the fact that our sampling is time-ordered, differently from the screening of the standard deviation or x-means clustering.^50^

The information about the movement of individual atoms needs to be converted to information about the movement of moieties. Besides using chemical intuition and the trivial knowledge of what atoms are directly connected, it is possible to assess the correlation between the movement of different atoms as the ratio between the intersection and the union of the frame numbers constituting their clusters. Clusters of values for individual coordinates for which such correlation is close to 1 are grouped into conformational basins (e.g., the clusters of all the dihedrals of the β-ionone ring are grouped into two conformational basins). The averaging of the internal coordinates is carried out in each conformational basin.

The movement of freely rotating groups (in our case only methyls) is independent, and consequently, the correlation, as measured above, is significantly lower than 1. The position of atoms of freely rotating groups is selected to be the center of the major cluster within that basin while enforcing a chemically sound structure. To do so, in the averaging of dihedral angles of freely rotating groups, the relative position of atoms is based on their average relative position during the trajectory within the basin. For instance, for a methyl group, one hydrogen is chosen, and its average dihedral angle is assigned as the center of its major cluster; then the two other hydrogens are placed to be  apart, where the exact value is given from the average over the trajectory within the basin. This suite of analytical tools was included in the *fdeta*(52) (FDET-Average) Python module.

### 2.2 Analysis of Basins

The similarity of two structures was measured by their minimized root mean-square deviation (Θ, cf. eq 19). In order to estimate how well a specific geometry represents an ensemble or a subensemble, sets of Θ values, , were used. In this case, *m* refers to the average structure, and *p* spans over the set of structures used to obtain the average one, i.e., the specific basin.

These sets of Θ values represent the distance from the average structure to all of those of the basin, and their distribution indicates the compactness of the basin.

The freely rotating groups would actually yield the largest contribution to Θ. In order to remove their contribution, *l* spans only a subset of atoms. Several choices are possible for such a subset: one is to only exclude methyl hydrogens; another possibility is to only use the β-ionone ring, excluding its methyl hydrogens. Excluding methyl hydrogens also avoids the problem of permutations of chemically equivalent atoms.

### 2.3 Computational Details

For this analysis, 4 previously investigated systems consisting of a protonated retinal Schiff base (subsystem A) in a protein environment (subsystem B) were considered. The systems differed in both the retinal configuration and the protein environment.

The first test case consists of the all-*trans* retinal chromophore embedded in the channelrhodopsin chimera C1C2,^53^ and we use 1 ns of a previously published quantum mechanics/molecular mechanics (QM/MM) molecular dynamics (MD) trajectory (monomer 1 with deprotonated residue E162).^54^ The QM region comprised the retinal chromophore as well as the K296, E129, K132, E162, and D292 side chains. The QM region was treated using SCC-DFTB,^55^ while the remaining protein and the membrane were described using the classical force field CHARMM36.^56,57^ This system is denoted as *AT-C1C2*. For full details of the computational setup, we refer to the original publication (ref (54)). We then turned our attention to the mutated jumping spider rhodpsin-1 (JSR1).^58−61^ We analyzed 1 ns QM/MM MD trajectories performed for the all-*trans*, 9-*cis*, and 11-*cis* configurations. The QM partition consisted of the retinal chromophore and K321 linking residue which were described using SCC-DFTB. The MM region comprised the remaining environment and protein which were calculated using the AMBER classical force field.^62^ We refer the reader to ref (58) for full details. The models from the work of Church et al. are, respectively, denoted by *AT-JSR1*, *9C-JSR1*, and *11C-JSR1*.

This system had been observed to have a significant spread in excitation energy (for our level of theory, it was in the 0.8–1.0 eV range). For all calculations, only the first excitation was considered, and frames were extracted in 1 ps steps. For *AT-C1C2*, the last 750 ps of the trajectory were used for our analysis, while for *JSR1*, the full trajectories were used. The selection of frames in all systems is the same as for the previous studies (cf. refs (54 and 58)).

In order to perform ensemble averaging, which will be our reference, two excited-state calculations per frame were performed: one in the presence of an embedding potential (*emb*) and one without (*iso*). The embedding potential, which is obtained from the linearized-FDET approximation,^63,64^ was obtained with Q-Chem 5.4’s^65^ implementation of Frozen Density Embedding Theory,^66^ using the Thomas-Fermi^67,68^ kinetic functional for , the Dirac–Slater^69^ functional for , and the Vosko-Wilk-Nusair^70^ parametrization of the LSDA correlation energy for . The Hartree–Fock density of the isolated protonated Schiff base was used as . The environment density ρ_*B*_ was obtained as the sum of the isolated Hartree–Fock densities of each residue having at least an atom within 5 Å of the retinal Schiff base, while residues between 5 and 12 Å away from the chromophore were modeled by means of their force field atomic charges, i.e. CHARMM36^56,57^ for *AT-C1C2* and Amber ff14SB^62^ for *JSR1*. Both the embedded and isolated excited state calculations were performed with the algebraic diagrammatic construction for the prepolarization propagator first order (ADC(1)), which yields the same excitation energies as configuration interaction singles but improved oscillator strengths.^71^ The cc-pVDZ^72^ basis set was used. The resolution of identity with the RIMP2-cc-pVDZ as auxiliary basis set was used.^73,74^ We decided to use a qualitatively, albeit not quantitatively, accurate method in order to investigate the accuracy of eq 17 and eq 18 in order to reduce the computational effort, considering that the focus of this article is the quality of the approximation in eq 17 and eq 18, rather than the comparison to experimental values.

The first excitation was then calculated at the same level of theory as the individual frames for each average chromophore structure both with and without an average embedding potential.

Since the averaging of the solvent to obtain the average embedding potential is done in Cartesian coordinates, we processed the aligned trajectory, obtained as outlined in subsection 2.1 (i.e., Kabsch alignment of subsystem A). The atoms in the environment were grouped by type(*t*) and modeled by means of their effective charge *q*_*i*(*t*)_. Their effective number of electrons is obtained as
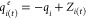
20where *Z*_*i*(*t*)_ is the nuclear charge of the atom *i*, of type *t*. For the sake of brevity, from now on, only *t* will be used. The average solvent density at the position of the grid point with index α (**r**_α_ = {*x*_α_, *y*_α_, *z*_α_}) is then evaluated as
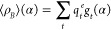
21where *g*_*t*_(α) is the time-averaged probability of finding a nucleus of type *t* in the volume element corresponding to the gridpoint α, divided by the volume ν_α_
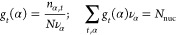
22

The average electrostatic potential is hence
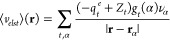
23

And finally, the embedding potential within linearized-FDET becomes
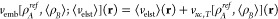
24

Only the frames pertaining to the *k*-th conformational basin used to obtain the solute structure were used to obtain the average embedding potential, guaranteeing to account for any coupling between the motion of the chromophore and of the environment.

The specific bonds used to calculate the bond length alternation (BLA) are shown in the Supporting Information. The range of the BLA values was then divided in 6 equal bins, and the frames were grouped based on which bin their BLA pertained to.

For random selection, the Python module *random*(75) was used. This uses the Mersenne Twister^76^ as its core generator.

## 3 Results and Discussion

The discussion of our numerical results will be organized as follows: subsection 3.1 will analyze and discuss the detection of the conformational basins and the related average structures, while subsection 3.2 will tackle the comparison of excitation energies and oscillator strengths from average structures to the ensemble of individual values.

Subsection 3.3 concerns the relation between a particular geometrical parameter (BLA) and the observable (excitation energy) in case of subsystem averaging.

### 3.1 Structures from Geometric Averaging

The major changes in conformation throughout the molecular dynamics simulation were detected by analysis of the trajectories of internal coordinates (cf. Section 2.3).

**Figure 1:**
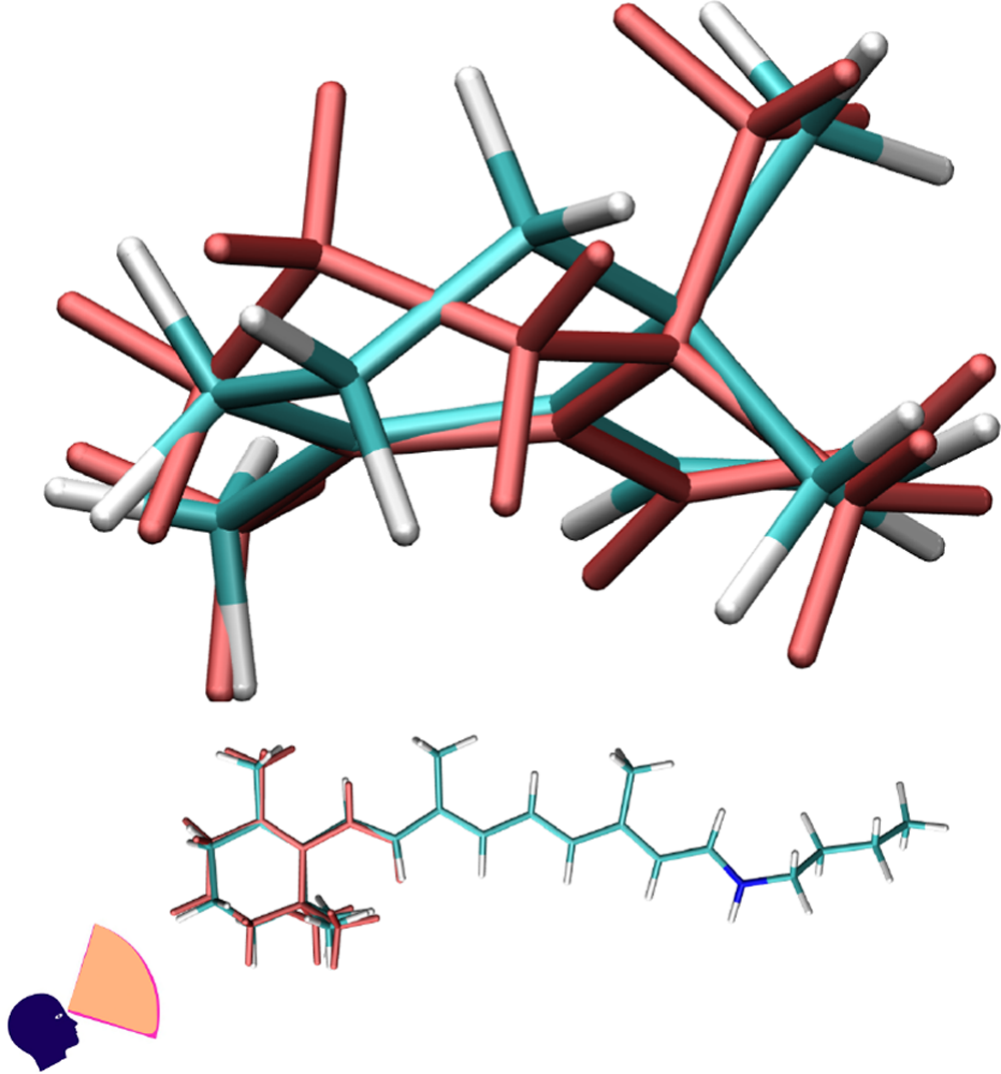
Comparison of  and  (light red) for *AT-C1C2*. The geometries’ centers have been superimposed, and alignment has been performed with the Kabsch algorithm.^47,48^

**Figure 2:**
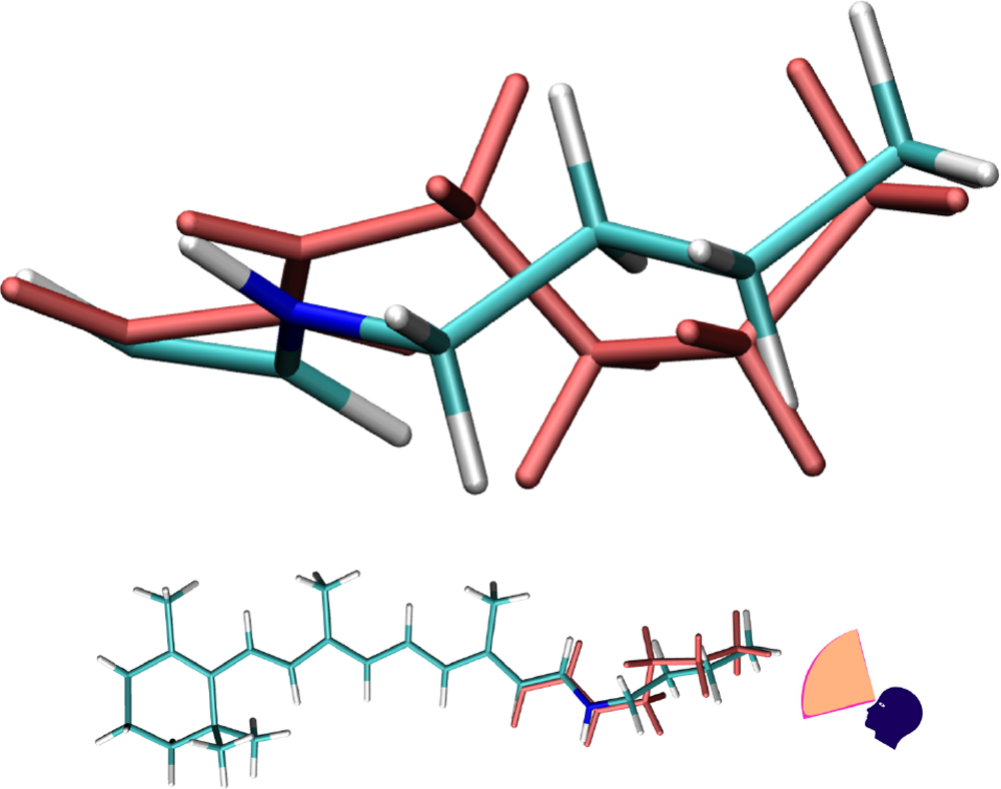
Comparison of superimposed  and  (light red) for *AT-C1C2*.

Two types of conformerism were detected, to different extents, in the different systems:

- the β-ionone ring is present in two possible conformations, as foreseeable (cf. Figure 1)
- the conformation of the lysine chain can fluctuate (cf. Figure 2)

Four conformations are expected due to the combined effect of these two motions, two to three of which were actually observed, depending on the system. In the cases of *AT-JSR1* and *11C-JSR1*, the less frequent conformation of the lysine chain amounted to only 15 and 21 frames, respectively. These conformational basins were hence ignored both because such a limited amount of frames may be an insufficient sample for proper averaging and because their weights in eq 17 are going to be minimal.

The detected conformational basins are identified as 1, 2, and 3 (when analyzed) with a decreasing number of frames pertaining to each conformational basin. Their related average structures are denoted by , , and . As a term of comparison, geometries produced ignoring the division into conformational basins were also produced. This method will be named “direct geometric averaging”, and its geometries will be denoted as .

A visual comparison of , , and  is given for *AT-C1C2* in Figure 1 and Figure 2, while similar figures are available for the other systems in the Supporting Information.

Generally, geometries from direct geometric averaging tend to resemble more  due to the larger number of frames associated with conformer 1, albeit they still tend to introduce some strain in the β-ionone ring, due to the mixing of the two conformations.

**Figure 3:**
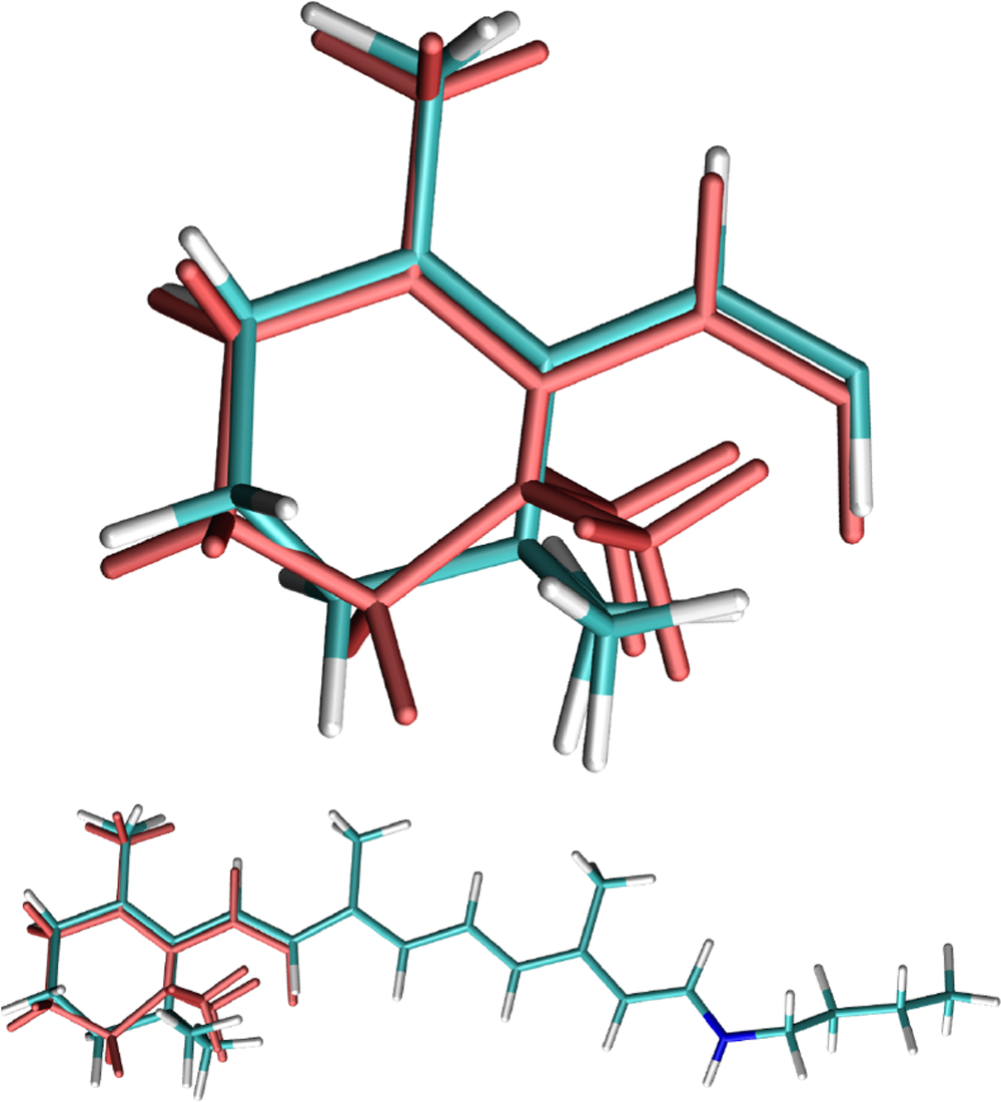
Comparison of superimposed  and  (light red) for *AT-C1C2*.

**Figure 4:**
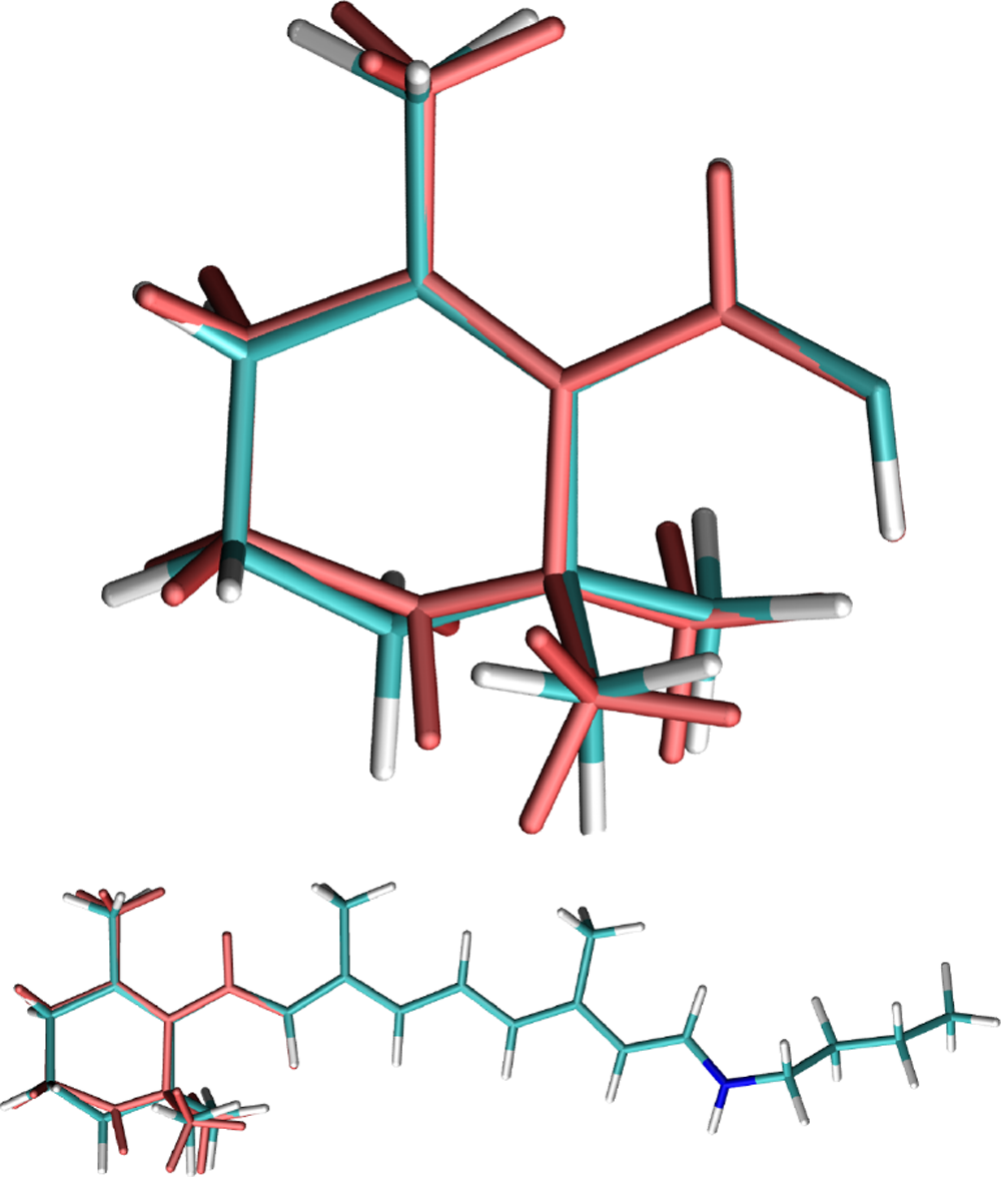
Comparison of superimposed  and  (light red) for *AT-C1C2*.

There are several possible ways to define connectivity in a module, resulting in a different conversion from Cartesian coordinates to internal coordinates. The common choice of defining the molecule as a chain from one end to the other may lead to error amplification, as any angle error results in a larger movement in the Cartesian space. This is even more important in the case of cycles, which are technically defined as chains, as the position of the last atom implicitly defines the last bond. In the case of the direct averaging of *AT-C1C2*, the use of the automatically defined connectivity - i.e. one where the β-ionone is defined as a single 6-carbon chain - leads to a geometry  with a significantly shortened C–C bond. In order to mitigate this problem, the connectivity was redefined so that the β-ionone is defined as a 4- and a 2-carbon chain, leading to . The visual comparison of these geometries to , clearly showing the shortened bonds, is in Figure 3 and Figure 4.

**Figure 5:**
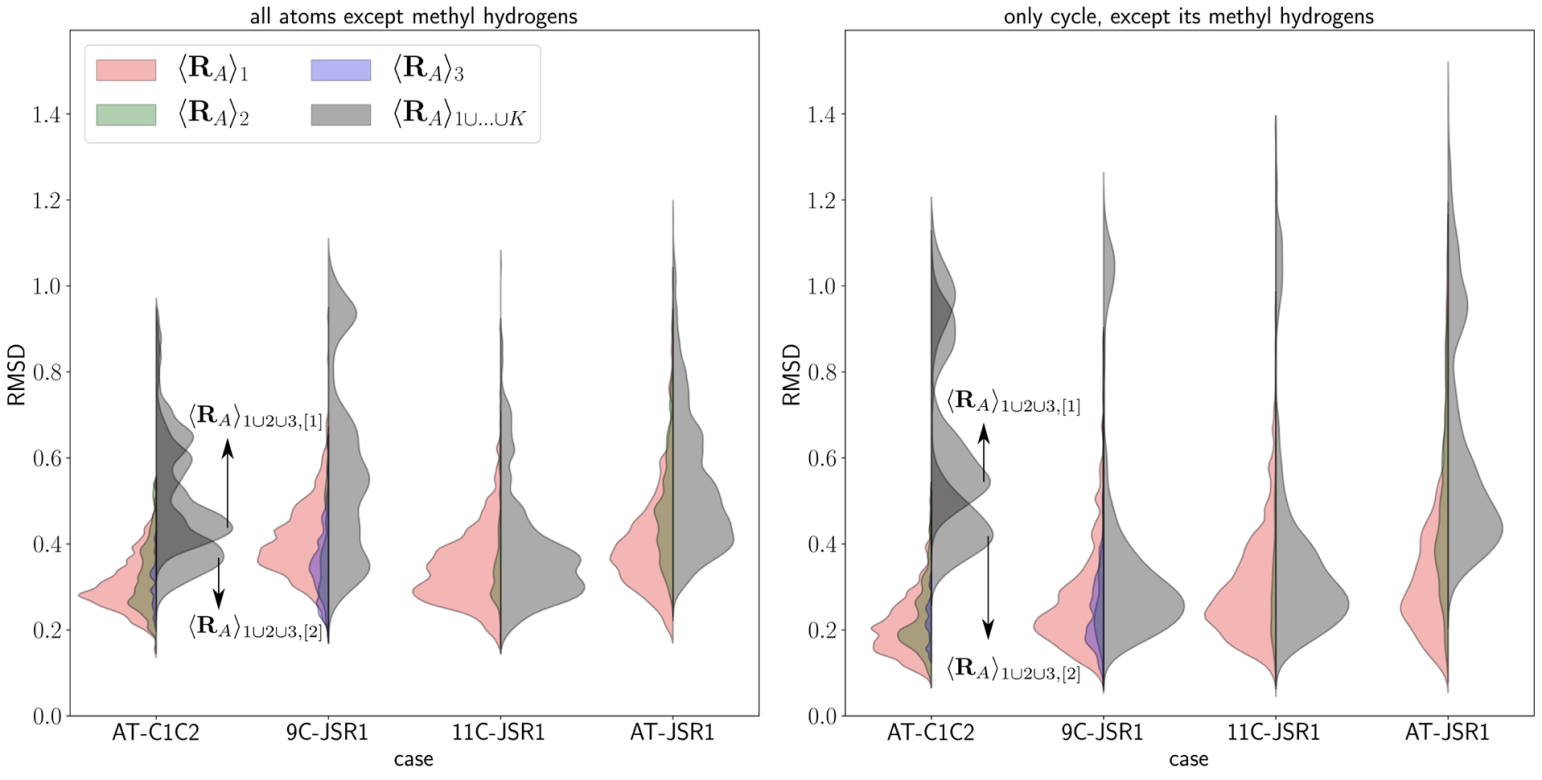
Distribution of Θ values (cf. eq 19) for basin-based (left half of the violins) and direct (right half of the violins) geometric averaging. In the left plot, *l* spans over all atoms but methyl hydrogens, while in the right one, it spans only on the β-ionone ring, with the exclusion of the methyl hydrogens.

A set of Θ values for each average geometry was computed, as outlined in subsection 2.2 by comparing it to all the frames used in the averaging procedure to obtain it. The distributions of these sets are shown in Figure 5, where in the left plot, the values of Θ are calculated using all atoms but methyl hydrogens, while on the right, only the β-ionone ring, excluding its methyl hydrogens, is used.

Figure 5 shows that basin-based average structures reliably constitute improvement over direct geometric averaging. This is highlighted by several different features of their distribution of Θ. First, the peaks of their distributions generally lie at lower Θ values than those of direct geometric averaging structures, and visual inspection of the curves clearly shows that any descriptor such as mean, median, distribution peak would be lower for basin-averaged structures. Second, their distribution curves tend to be more narrow than those of direct geometric averaging structures, and their distribution curves show a single peak (albeit with shoulders); while those of direct geometric averaging structures show multiple peaks pertaining to the different basins. Even if this feature is sometimes not so pronounced if Θ is calculated for the whole molecule with the only exception of methyl hydrogens, it is always clear when the Θ calculation is restricted to the moiety involved in the conformerism.

It is worth mentioning that the distribution of Θ or analogous similarity parameters is a powerful tool to test the validity of averaged structures because any reasonably important conformational basin that has not been detected will result in a separate peak.

It is also worth noticing that when restricting the calculation of Θ to the sole β-ionone ring, the distributions for basin-based structures tend to shift to slightly lower values, showing that the β-ionone ring is not the main contributor to the Θ values. On the contrary, the peaks of distributions from direct geometric averaging (or some of the peaks when the effect of the lysine tail is significant) shift to higher values, showing that the β-ionone atoms are actually the largest contributor in the summation to obtain Θ. This feature may, in the future, be used to make the production of basin-based structures automatically, by means of iterative divisions of the trajectory based on peak recognition in the distribution of Θ.

**Figure 6:**
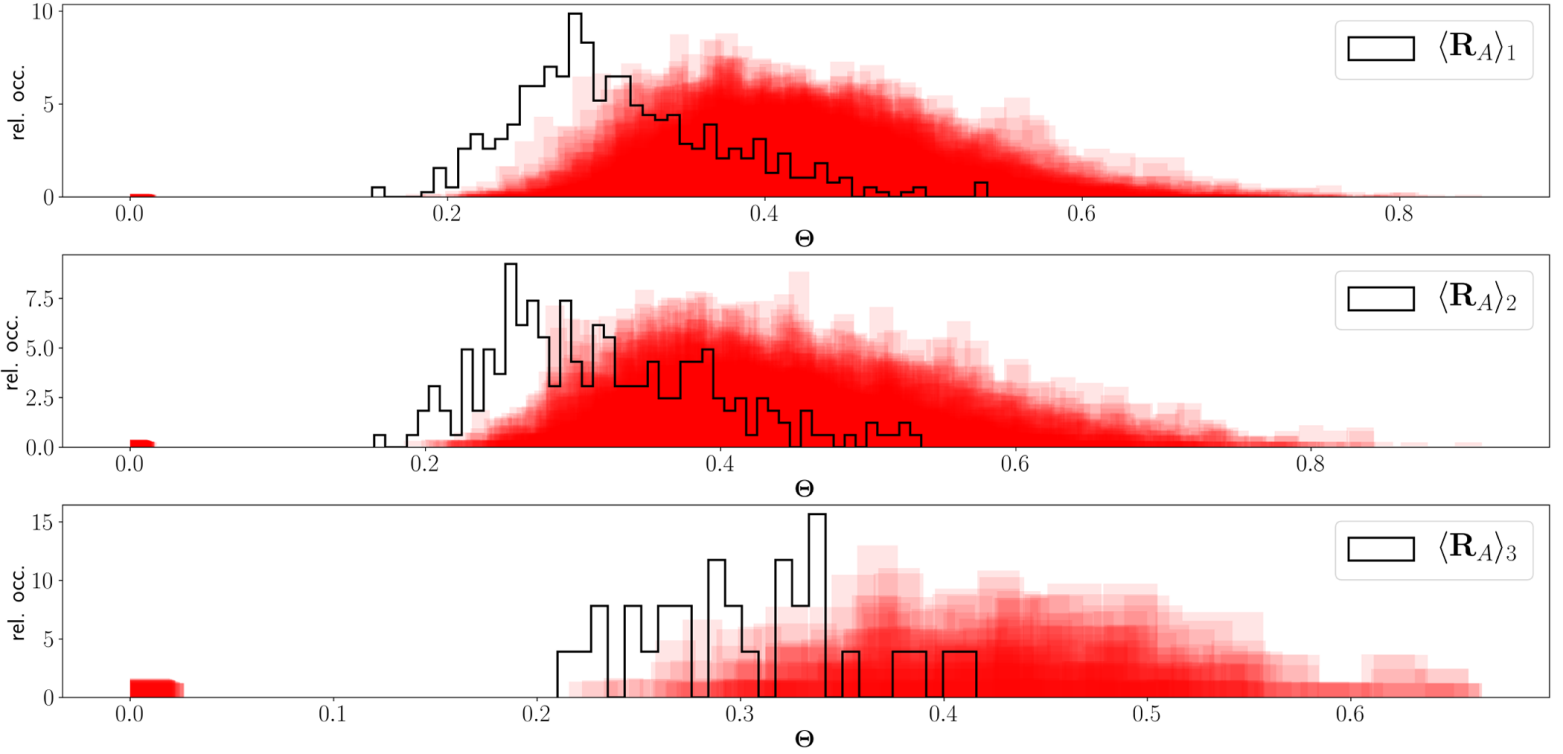
Distribution of Θ values (cf. eq 19), calculated for all atoms except methyl hydrogens, for each average structure (black line) and for randomly selected frames (low opacity red bars) for *AT-C1C2*. 75 random frames were used when possible, while the whole basin (31 frames) was used for 3. “rel. occ.” denotes the relative occurrence.

Below, we take a closer look at the basin-averaged structures and actual geometries taken from the trajectory. Considering Θ as a measure of the distance between two structures, a properly averaged structure should have a more favorable distribution of Θ than any of those used to obtain it. Nonetheless, considering that the averaging process is far from trivial, this is an important feature to confirm. To investigate this, 75 random frames were selected from each basin, and their distributions of Θ were compared to that of the basin’s averaged structure. As outlined in subsection 2.2, Θ is calculated only between a selected structure and the frames from the conformational basin it belongs to. This is shown in Figure 6. Analogous figures for the other systems are available in the Supporting Information.

Beside a small cluster around zero, due to the randomly selected geometry itself and a few extremely similar ones, all of the distributions of Θ from random frames lie to the right of that of the average structure. This clearly shows that the basin-based geometric averaging procedure is reasonable, and it is better to use basin-averaged structures than a randomly selected structure per basin.

Despite the averaging procedure being performed without direct use of information on the thermodynamics of the system, it is indirectly determined by them, provided the sampling is sufficient: the clusters of values for internal coordinates relate to convex regions of the free energy surface, hence the name “basin”. Consequently, considering that the sampling of the ensemble is performed at a specific temperature and for the full system, our coordinate averaging and the use of minimization of free energy of the supersystem can actually be considered related.

Since the division of the trajectory in *K* conformational basins, corresponding to the minimum number of structures to represent the trajectory, is dictated by the thermodynamics of the system, the computational savings are system-dependent. For specific highly flexible systems, the computational savings may potentially be relatively modest. It is nonetheless reasonable to expect a small value of *K* and hence a large computational advantage for most systems, specifically when embedded in soft condensed matter such as enzymes rather than in liquids.

### 3.2 Observables from Ensemble and Geometric Averaging

The relationship between a coordinate and a selected observable is far from trivial. Consequently, the sensitivity of an observable on a similarity parameter, such as Θ, cannot be foreseen. Nonetheless, there is no guarantee that a poorly representative structure will yield good observables.

**Figure 7:**
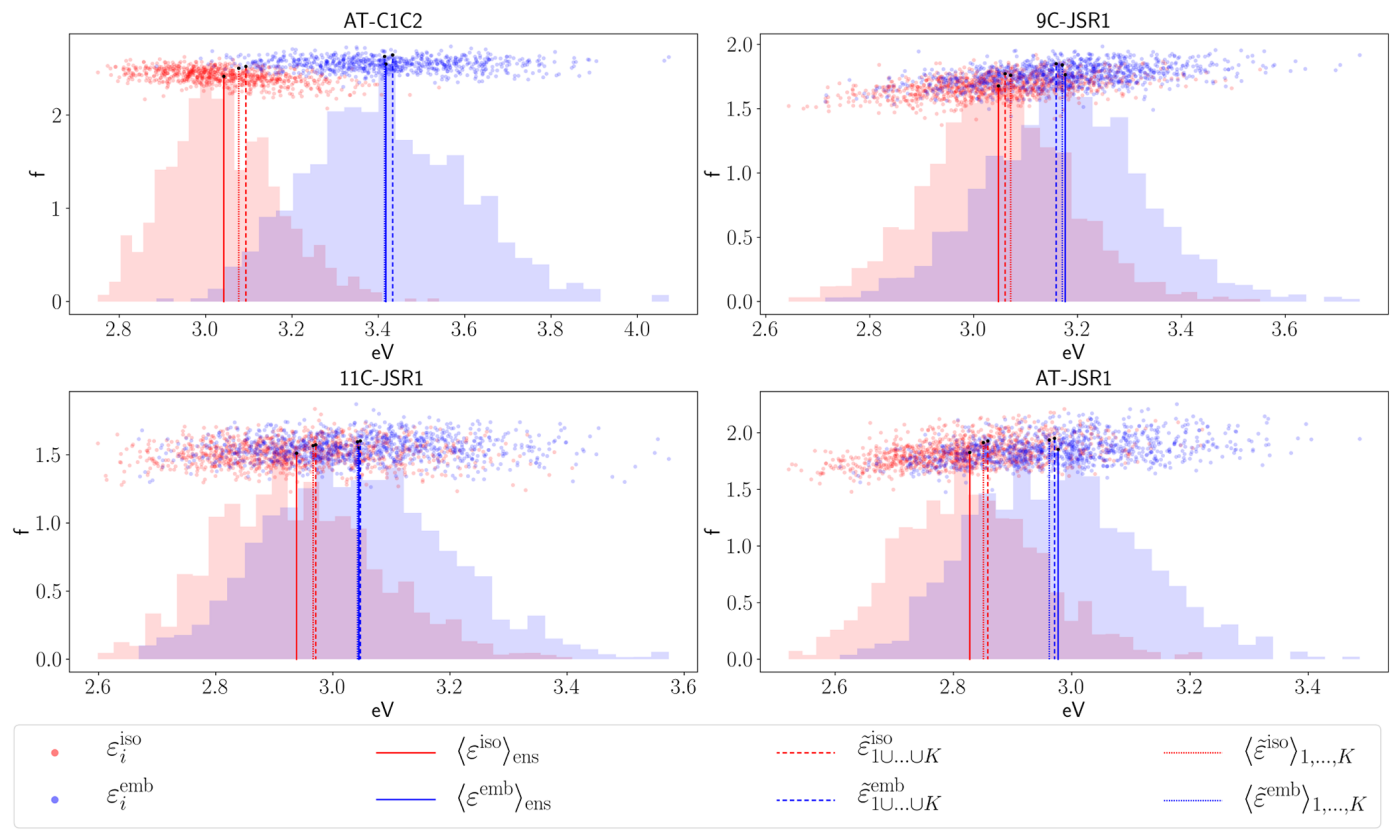
Ensemble, basin-based geometric, and direct geometric averaging for all systems. Legend refers to ε but also applies to *f*. The red and blue with low opacity represent the oscillator-weighted distribution of excitation energies for the iso and emb, respectively. The distributions are normalized so that their maximum matches the oscillator strength of the ensemble averaged value. For *AT-C1C2*, only  is shown. The same picture but including the value for  is available in the Supporting Information.

After we examined the average solute structures and compared them to those from direct geometric averaging and to randomly selected frames, we can turn to physical observables. We focus our interest in excited states properties, so the first excitation energy and its associated oscillator strength were calculated with ensemble and geometric averaging for every conformational basin, according to eq 15 and eq 16, and then the weighted averages were obtained according to eq 17 and eq 18. These excitation energies are shown in Table 1 and Table 2, respectively, while the oscillator strengths are available in the Supporting Information. Table 2 constitutes our principal result. A comparison of the ensemble of observables, ensemble averaged observables, and direct and basin-based geometric averaging is available in Figure 7 (all basins combined) and in the Supporting Information (individual basins).

**Table 1:** Excitation Energies from Ensemble Averaging ⟨ε⟩_*k*_ vs Geometric Averaging for Single Geometries  (Respectively Left and Right Hand Side of Eq 15)^a^

system	*k*	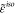	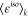	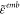	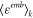	Δε^*iso*^	Δε^*emb*^	*N*_*frames*_
*AT-C1C2*	1	3.074	3.035	3.410	3.407	0.039	0.003	497
*AT-C1C2*	2	3.082	3.059	3.415	3.437	0.023	–0.022	219
*AT-C1C2*	3	3.080	3.047	3.478	3.469	0.032	0.009	31
*AT-C1C2*	1 ∪ 2 ∪ 3, [1]	2.595	3.042	3.044	3.417	–0.447	–0.373	750
*AT-C1C2*	1 ∪ 2 ∪ 3, [2]	3.093	3.042	3.433	3.417	0.051	0.016	750
*9C-JSR1*	1	3.074	3.049	3.174	3.176	0.025	–0.002	691
*9C-JSR1*	2	3.073	3.054	3.180	3.186	0.019	–0.006	165
*9C-JSR1*	3	3.053	3.036	3.134	3.162	0.017	–0.028	99
*9C-JSR1*	1 ∪ 2 ∪ 3	3.061	3.048	3.159	3.176	0.013	–0.017	1000
*11C-JSR1*	1	2.973	2.941	3.048	3.047	0.033	0.001	863
*11C-JSR1*	2	2.882	2.898	2.964	3.011	–0.016	–0.047	69
*11C-JSR1*	1 ∪ 2	2.971	2.938	3.047	3.044	0.033	0.002	1000
*AT-JSR1*	1	2.832	2.811	2.943	2.960	0.020	–0.017	741
*AT-JSR1*	2	2.904	2.870	3.016	3.023	0.034	–0.007	244
*AT-JSR1*	1 ∪ 2	2.858	2.828	2.971	2.977	0.031	–0.006	1000

a Errors are defined as . Excitation energies and errors are given in eV. *N*_*frames*_ denotes the total number of frames in the basin.

**Table 2:** Excitation Energies from Ensemble Averaging ⟨ε⟩_*ens*_ (Cf. Eq 13) vs Geometric Averaging for the Weighted Average of Basins  (Cf. Eq 17)^a^

system	*k*	⟨ε̃iso⟩	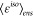	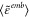	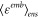	Δε^*iso*^	Δε^*emb*^
*AT-C1C2*	1,2,3	3.077	3.042	3.414	3.417	0.035	–0.003
*9C-JSR1*	1,2,3	3.071	3.048	3.171	3.176	0.024	–0.005
*11C-JSR1*	1,2	2.966	2.938	3.042	3.044	0.028	–0.002
*AT-JSR1*	1,2	2.851	2.828	2.962	2.977	0.023	–0.015

a Errors are defined as . All values are in eV.

Methods based on the self-consistent optimization of the solute and solvent such as ASEP/MD^7,8^ and ASEC-FEG^9,12,14,16,17^ require geometric convergence of the average structures of the solute and of the solvent for each optimized conformation, requiring  molecular dynamics simulations and  quantum mechanical calculations, where *N*_cycles_(*k*) is the number of cycles necessary for geometric convergence for conformer *k*. Conversely, our approach requires a single sampling of the statistical ensemble via molecular dynamics or molecular Monte Carlo and *K* quantum mechanical calculations.

The excitation energy and oscillator strength values obtained from ensemble averaging (⟨ε⟩_*ens*_, ⟨*f*⟩_*ens*_) sit in the center of the scatter of the individual values ε_*i*_, as would be expected (cf. Figure 7). Bootstrapped errors have been produced, and their size relates to the number of frames in each basin, as foreseeable. For the ensemble averaging over the whole trajectories, the error bars cannot be visually distinguished, and have hence been omitted in Figure 7, but are available in the Supporting Information.

The values from *AT-C1C2*’s  stand out as qualitatively wrong (cf. Table 1). This can be ascribed to the aforementioned artificially shortened bond, which also happens to be close to the conjugation chain, causing a larger destabilization in the excited state than in the ground-state, significantly lowering the excitation energy. This is not the case for  (cf. Table 1, Figure 7).

All direct geometric averaging values , both isolated (*iso*) and embedded (*emb*), except that of , are very similar to those from basin-based geometric averaging. This is, although specific to our test cases, where the moieties leading to conformerism are not involved in the excitation and appear not to influence it. Basin-based averaging ought to be preferred nonetheless, as it can be expected to be more robust since it prevents unphysical combinations of dihedrals and angles and avoids unphysical undefined bonds such as for . Furthermore, other systems may have conformational basins that strongly influence the chosen observables, either directly via geometric effects or indirectly if the movement of the environment is coupled to the conformerism, potentially introducing significantly larger errors in the embedded calculation than in the isolated one, affecting the magnitude of the calculated solvatochromic shift.

The values for the isolated solute (*iso*) from basin-based geometric averaging within a single basin  seem in good agreement with those from ensemble averaging  (cf. Table 1). Basin-based geometric averaging seems to almost consistently (in all but one case) overestimate the excitation energy. At the current state, this cannot be thoroughly explained. Further analyses ought to assess whether this is due to an excessively low ground-state or an excessively high excited state. On the other hand, this is not a straightforward task, as ground-state energies are strongly stabilized by having all 5 methyl groups in a favorable position, which in turn should not affect excitation energies significantly. Since the error is very similar in all basins, the weighted averages display the very same trend (cf. Table 2).

The embedded solute values (*emb*) from geometric averaging, , within a single basin also seem in good agreement with those from ensemble averaging, . Albeit in some cases almost equal to the reference ensemble averaging value , the error varies more than for the isolated solute (*iso*). There appears to be a slight trend to underestimate the excitation energy. This trend influences the weighted average  to an extent which is determined by the weight of the basin with the largest error. As a result, the weighted average  has negative errors which are smaller in magnitude than those for , often by a factor 10 (cf. Table 2). The net effect is that the solvatochromic shifts are slightly underestimated. Specifically, errors on solvatochromic shifts range for individual basins from −23.0 to −45.5 meV, with a mean absolute error of 34.1 meV, and a mean relative error of 21%. Further investigation is necessary to understand to what extent this trend is general to other systems and what causes it.

Our analysis of FDET-based calculations with averaged liquid environments showed a deterioration of the results below a certain number of frames.^18^ Such a threshold cannot be directly applied to drastically different systems, such as protein environments, where the movement may be different in the entity and speed. An excessively low number of frames may be a necessary but not sufficient condition for a relatively large error. If some kind of correlation between the number of frames used to average and the quality of the results was confirmed, one may choose to use a fixed number of frames to average within each basin. In such a case, the weights, which in this work are obtained from the populations of the subensembles, could also be obtained with some other method such as free energy perturbation theory.

Alternatively to geometric averaging, a random selection of frames may be used to reduce the number of calculations to perform. The use of the random sampling method has been exemplified on the photoactive yellow protein by Isborn and co-workers.^77^ These authors found that the statistical independence of each frame was related to the thermostat and time step of the trajectory. In order to compare these two strategies, we determine how many randomly selected frames are necessary to ensure, with a certain confidence, that the error from the random selection is lower than that from geometric averaging. To determine such a number, denoted as *s*_*c*_, an ensemble averaged observable is calculated for incrementally larger sets of random frames. Specifically, we perform the following procedure for excitation energy ε.

- we start with an empty set of selected frame indices .
- for *s* ranging from 1 to the total number of frames, *N*, we randomly sample a frame *i*′ ∉*I*_*s*_ and obtain *I*_*s*_ = *I*_*s*–1_ + *i*′.
- as a result, we obtain the set of *N* incrementally selected subsensembles *I*_*s*_. Each subensemble *I*_*s*_ leads to a value of , all of which are collected in the *N*-valued set .
- the previous steps are repeated for *r* ranging from 1 to *R*, obtaining
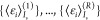
25
- the division into sets is reorganized as , and for each of these sets, the mean and standard deviation are calculated:
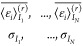
26
- we obtain the interval from  to  which represents the confidence interval for . The choice of an amplitude  is due to the fact in the case of a normal distribution it would constitute a 95% confidence interval.

**Figure 8:**
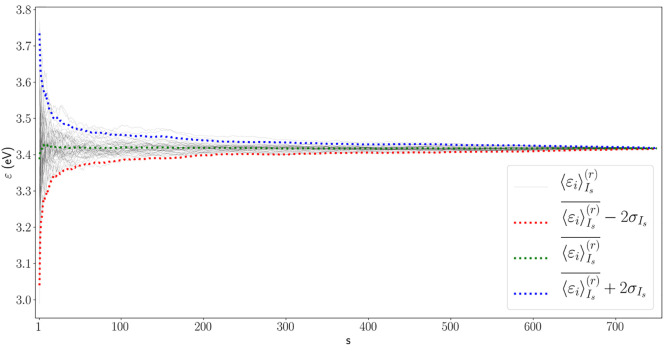
Evolution of the ensemble averaged excitation energy  (cf. eq 25) of embedded *AT-C1C2* for incrementally larger randomly selected subensembles (full black lines). 50 such lines are represented (*r* = 1, ..., 50). Their average  (cf. eq 26) is shown as a dashed green line. The lower  and upper  limits of the confidence interval are shown as red and blue dashed lines, respectively.

The results of such a procedure are shown in Figure 8 for the case of embedded *AT-C1C2*, with R = 50. By construction, the values for *s* = 1 correpond to randomly selected single frames, while for *s* = *N*, we have the full ensemble, regardless of *r*:
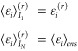
27The confidence interval, as expected, decreases in magnitude until it is zero for *s* = *N*. When *R* is increased, the curves for the limits of the confidence interval, and its center, become smoother. This is shown in the Supporting Information for R = 100, 1000. Analogous figures for the other system are qualitatively similar.

Finally, we can determine *s*_*c*_ as the value for which the confidence interval no longer comprises the result from geometric averaging . Such a value represents the number of randomly selected frames to be, with an ∼95% confidence, more accurate than . Such values are shown in Table 3. The smaller , the more frames will be necessary for the confidence interval to shrink sufficiently, i.e. the larger *s*_*c*_ will be. This is the reason for the generally larger values of *s*_*c*_ for *emb*: Δε^*emb*^ values are 1 order of magnitude smaller than Δε^*iso*^ (cf. Table 2). The necessary random frames *s*_*c*_ are at least 1 order of magnitude more than the 2–3 frames necessary for geometric averaging.

**Table 3:** Number of Randomly Selected Frames *s*_*c*_ so That the  Confidence Interval Does Not Include the Value from Geometric Averaging a

	*s*_*c*_	
system	*iso*	*emb*
*AT-C1C2*	46	707
*9C-JSR1*	121	781
*11C-JSR1*	80	938
*AT-JSR1*	89	279

a The random selection has been performed *r* = 1000 times to obtain the confidence intervals.

### 3.3 Bond Length Alternation and Its Effect on Excitation Energy

**Figure 9:**
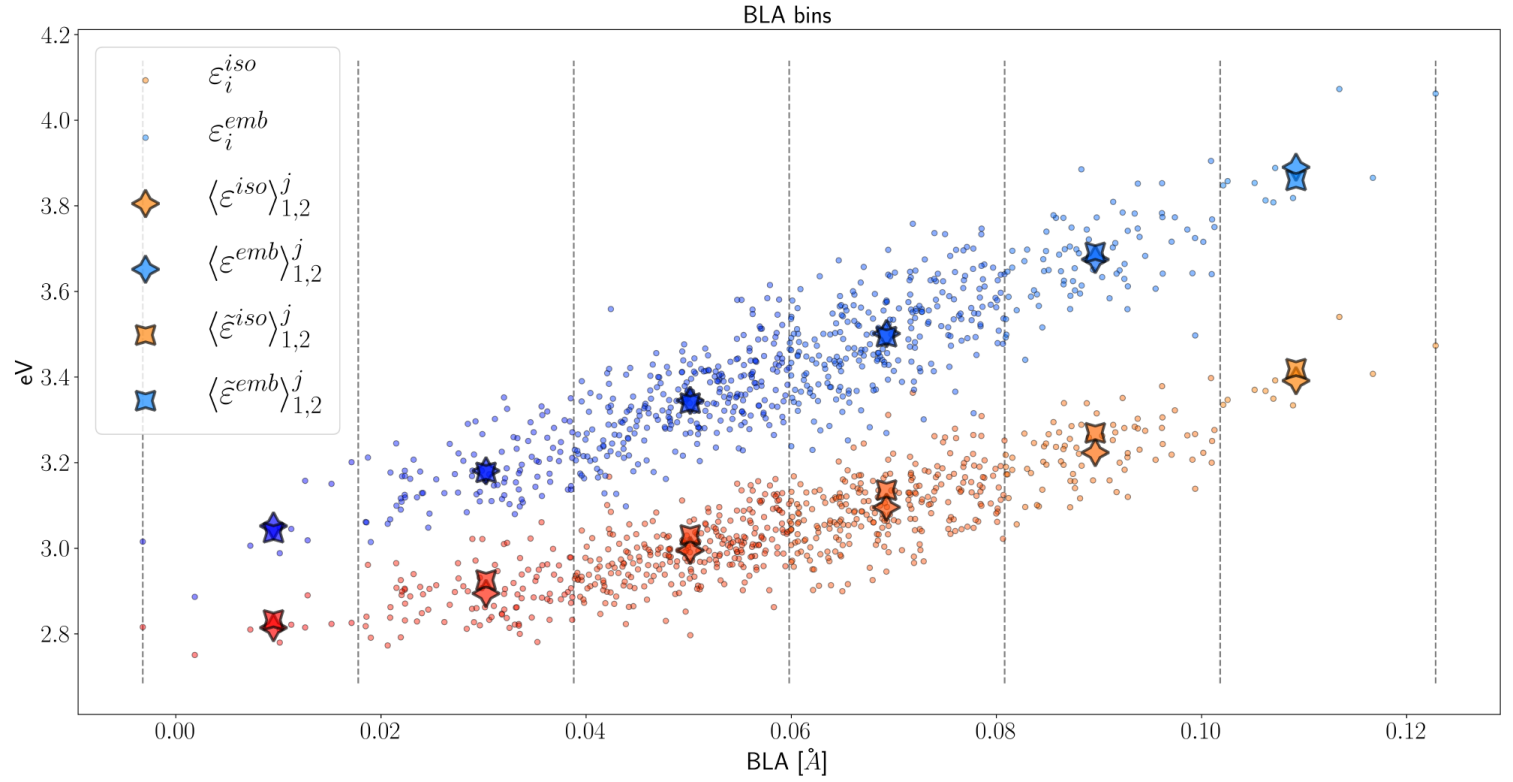
Ensemble vs average geometry, weighted for conformers 1 and 2, within 6 BLA-based bins. Conformer 3 was neglected in this case. The superscript *j* in the legend denotes that ensemble and basin-based geometric averaging are restricted to the *j*-th bin.

In order to test the assessment of geometry-observable relations by means of basin-based geometric averaging, we considered the bond length alternation (BLA) as a collective independent variable. BLA, defined as the difference between the average single bond and the average double bond, has been found to be linearly related to the excitation energies in conjugated polymers.^78−80^ This is the case for our retinal Schiff base, as was observed specifically in *AT-C1C2* in ref (81), as can be seen from the scatter of individual values in Figure 9 (numerical values available in the Supporting Information). As expected, ensemble averaging within each subensemble yields the same trend as that of the full scatter. Basin-based geometric averaging was performed in 6 equally spaced BLA-based bins, each divided in 2 based on the β-ionone ring conformation, while the 31 frames due to the lysine tail’s conformerism have been discarded to this end. The excitation energies from basin-based geometric averaging are not exactly equal to those from subensemble averaging but clearly yield a comparable trend, both for isolated (*iso*) and embedded solute (*emb*). Namely, there is a clear linear correlation between BLA and excitation energy, with its linear coefficient being slightly larger for the embedded chromophore.

## 4 Conclusions

We introduce an approach for the analysis of internal coordinates to detect conformational basins within which the internal coordinates are averaged. The soundness of the division into basins was assessed by observing the distribution of the minimized root-mean square deviation (Θ) of the average structure to those that it averages. These distributions are single-peaked.

Observables for the whole ensemble can be modeled as the average of observables for each average structure with small errors. This paves the way for the extension of environment averaging to systems where multiple conformations are thermally accessible and leads to large computational savings: instead of *N* quantum calculations, where *N* is the number of samples of the statistical ensembles, *K* calculations were performed, where *K* is the number of detected conformational basins, in our case 2–3.

Another advantage of the proposed procedure is that it does not require self-consistent optimization of the solute and its environment, which, for each basin, would require multiple sampling procedures and quantum calculations. Only one sampling is required for the full ensemble, and only one quantum calculation is performed per basin. The reduction in the required number of calculations may allow the analysis of longer trajectories or larger quantum regions or the use of higher level electronic structure methods.

Lastly, we applied the same procedure to arbitrary subensembles in order to assess the relation between the geometrical parameters and observables.

The concept that this manuscript hinges on, namely that a subensemble of similar solute structures can be represented by its average, may be applied with protocols differing in the clustering techniques, in the observables analyzed, in the type of solute used, and so on and so forth. Albeit thorough analyses of these specific aspects are necessary, we show the importance and the soundness of the main approximation.

## Supporting Information Available

Visual comparison of the average solute structures for the systems not shown in this manuscript, distribution of the Θ values for randomly selected frames for the systems not shown in this manuscript, a table with the oscillator strength values for ensemble and geometric averaging, spectra for individual basins for each system, and numerical values related to Figure 9 (PDF)
