# Supplementary material for: Embedding Nonrigid Solutes in an Averaged Environment: A Case Study on Rhodopsins
Source: J Chem Theory Comput. 2023 Jul 13;19(15):5289–302. doi: 10.1021/acs.jctc.3c00285 (PMC10413860; doi:10.1021/acs.jctc.3c00285)
Supplement: Supplementary file 1 — ct3c00285_si_001.pdf [file ct3c00285_si_001.pdf]

Supporting Information of: Embedding flexible solutes in an averaged environment

Niccolò Ricardi,*,† Cristina E. González-Espinoza,† Suliman Adam,‡ Jonathan Church,‡ Igor Schapiro,*,‡ and Tomasz Adam Wesołowski*,†

†*Department of Physical Chemistry, University of Geneva, Geneva (Switzerland)*

‡*Fritz Haber Center for Molecular Dynamics, Hebrew University of Jerusalem Israel, Jerusalem (Israel)*

E-mail: Niccolo.Ricardi@unige.ch; Igor.Schapiro@mail.huji.ac.il; Tomasz.Wesolowski@unige.ch

S1 Basin-based averaging

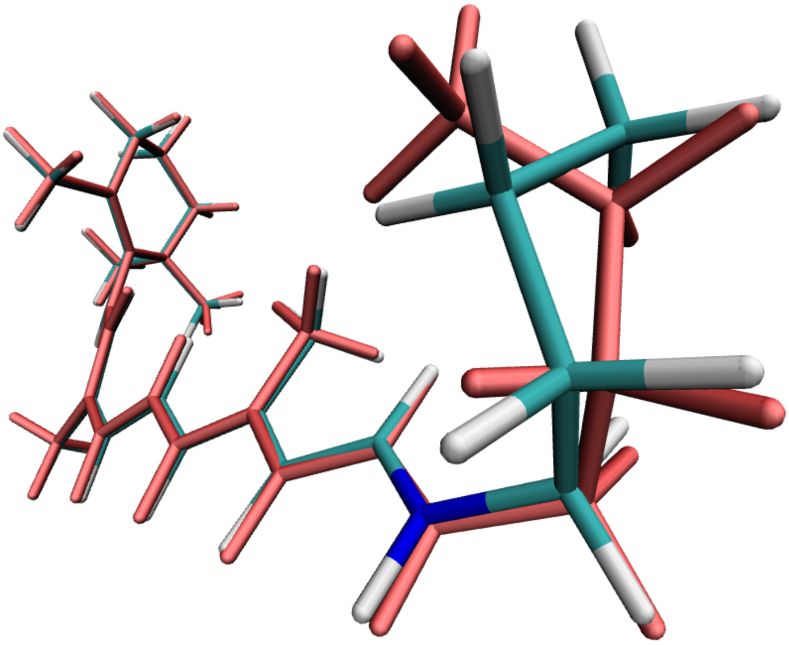

Figure S1: Comparison of $\langle \mathbf{R}_A \rangle_1$ (red) and $\langle \mathbf{R}_A \rangle_2$ (light blue) for *9C-JSR1*. The geometries' centers have been superimposed and alignment has been performed with the Kabsch algorithm.

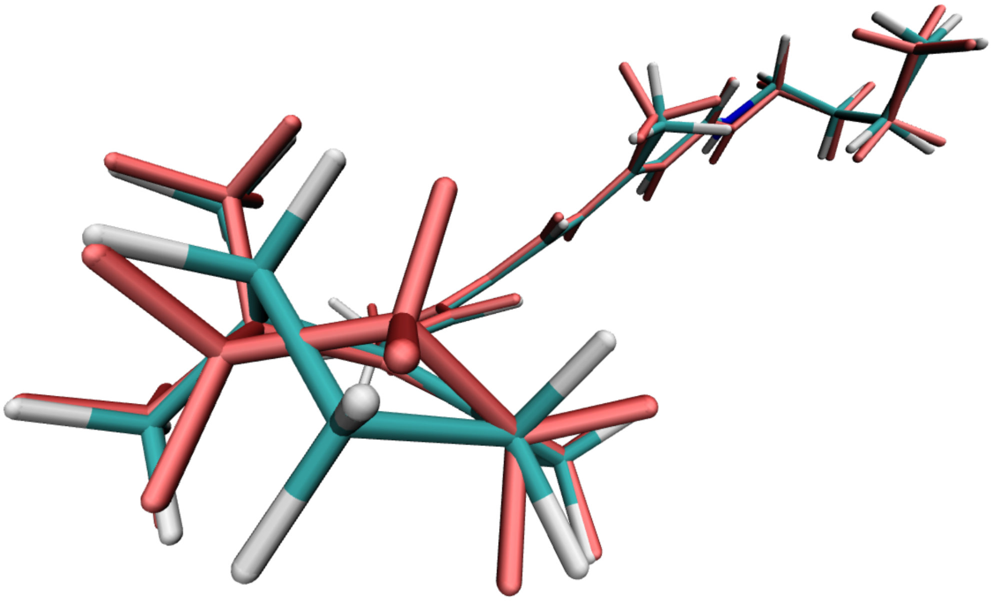

Figure S2: Comparison of $\langle \mathbf{R}_A \rangle_1$ (red) and $\langle \mathbf{R}_A \rangle_3$ (light blue) for *9C-JSR1*.

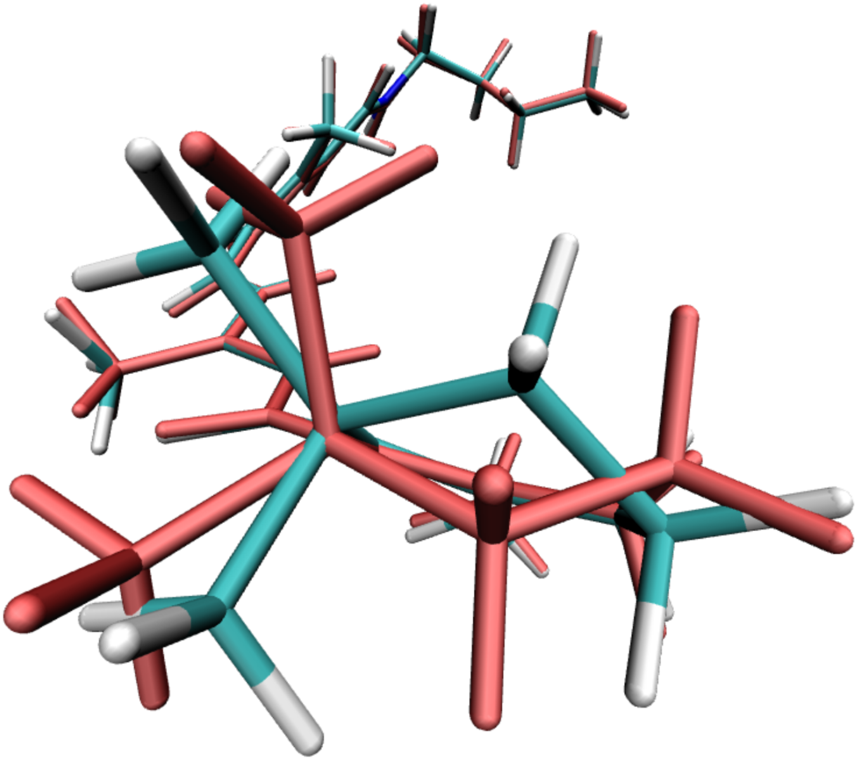

Figure S3: Comparison of $\langle \mathbf{R}_A \rangle_1$ (red) and $\langle \mathbf{R}_A \rangle_2$ (light blue) for *11C-JSR1*.

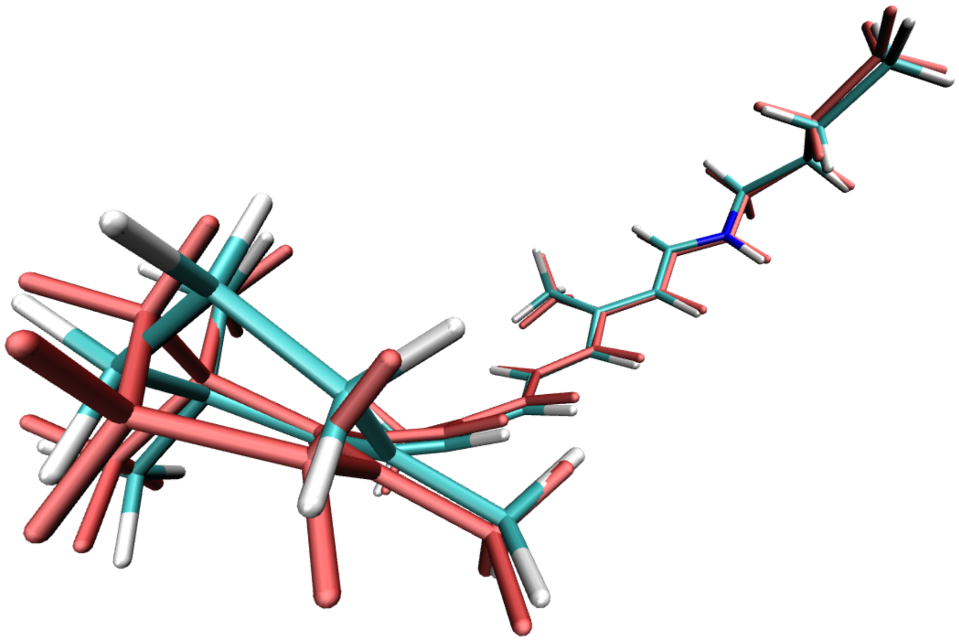

Figure S4: Comparison of $\langle \mathbf{R}_A \rangle_1$ (red) and $\langle \mathbf{R}_A \rangle_2$ (light blue) for *AT-JSR1*.

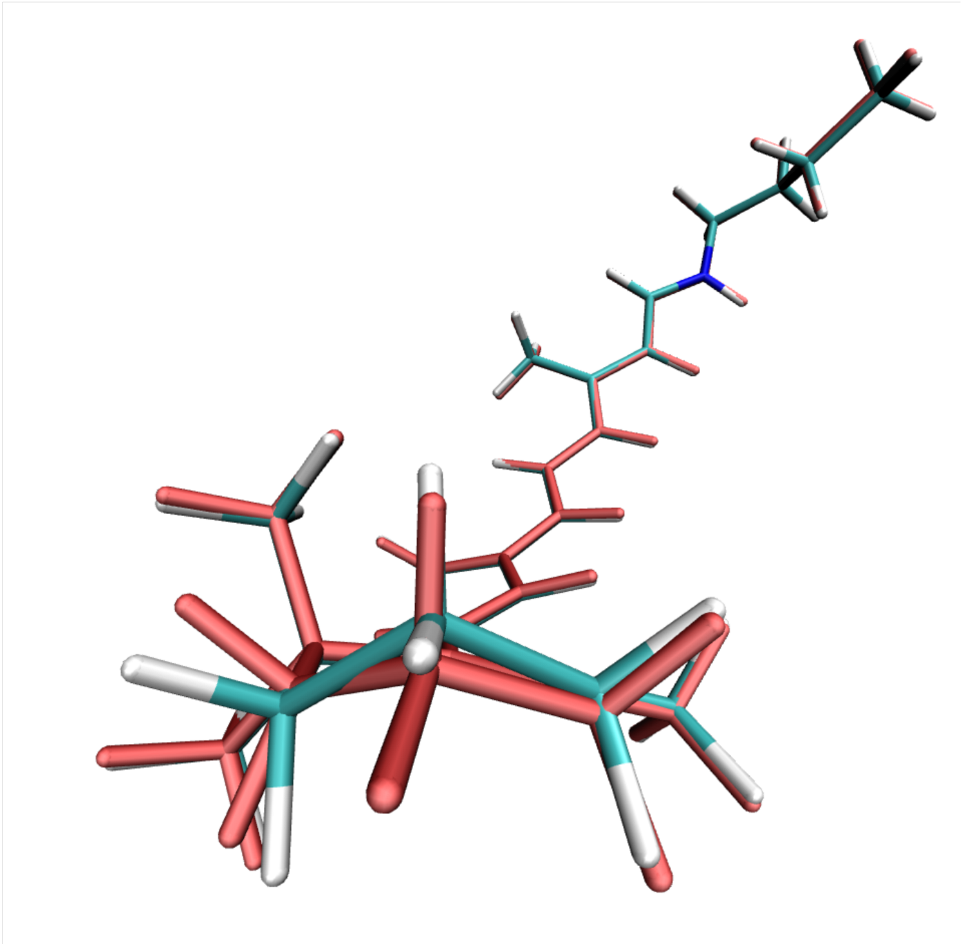

Figure S5: Comparison of $\langle \mathbf{R}_A \rangle_1$ (red) and $\langle \mathbf{R}_A \rangle_{1\cup 2}$ (light blue) for *AT-JSR1*.

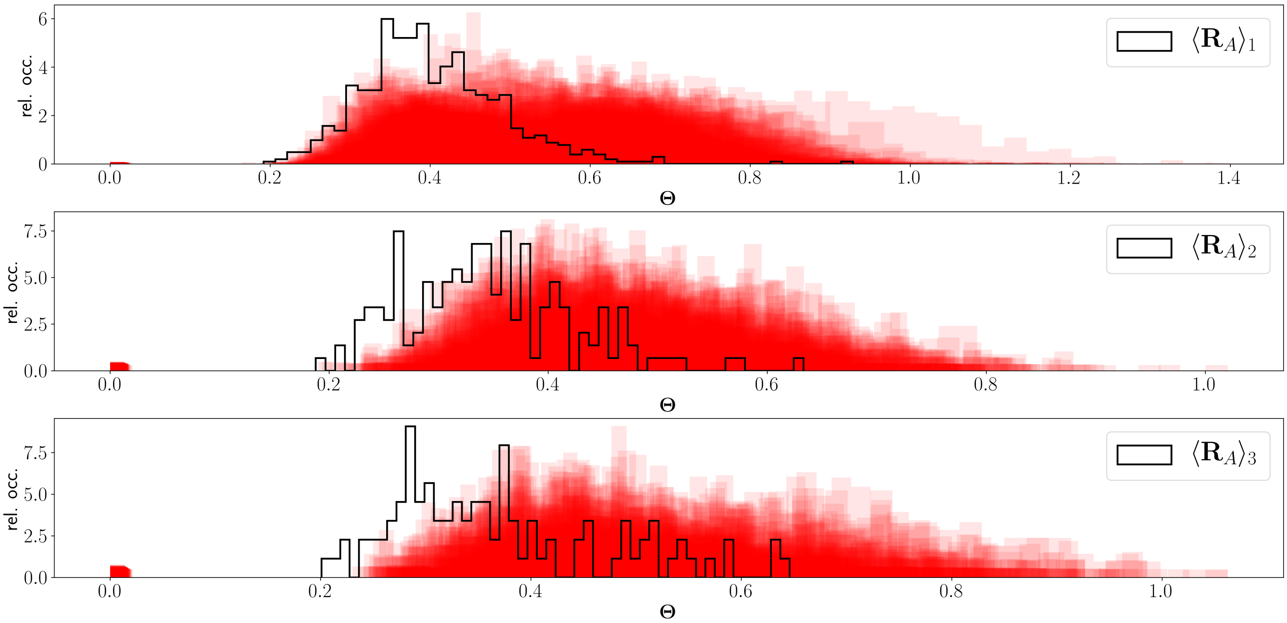

Figure S6: Distribution of Θ values, calculated for all atoms but methyl hydrogens, for each average structure (black line) and for 75 randomly selected frames (low transparency red bars) for *9C-JSR1*.

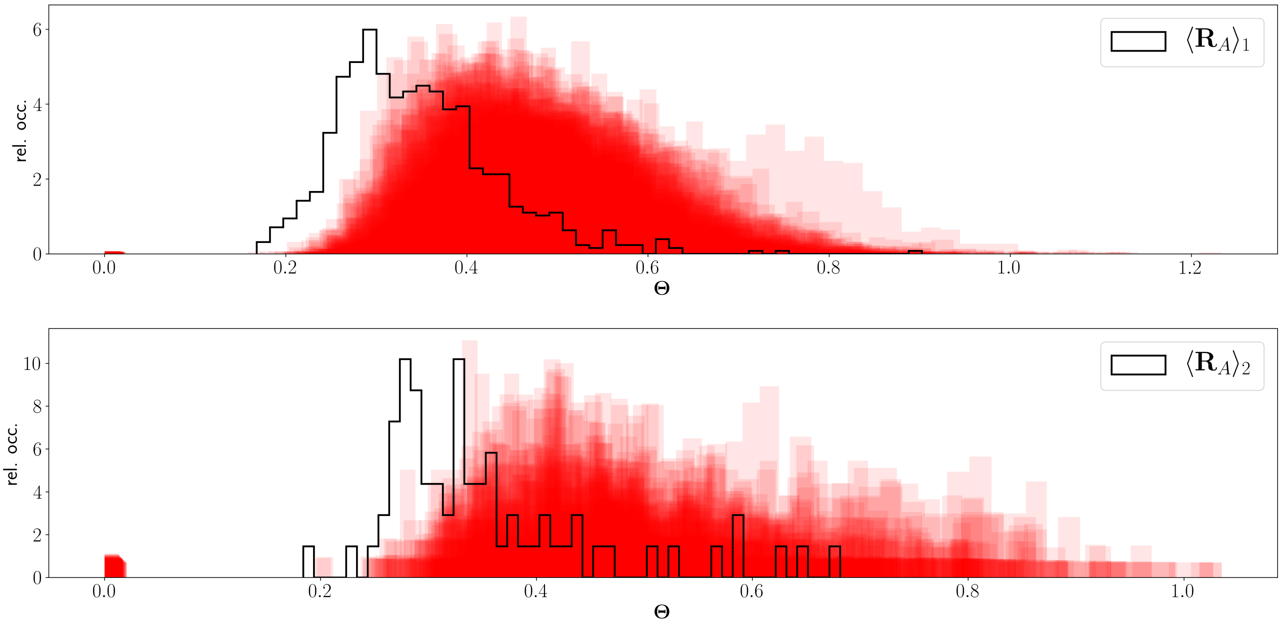

Figure S7: Distribution of Θ values, calculated for all atoms but methyl hydrogens, for each average structure (black line) and randomly selected frames (low transparency red bars) for *11C-JSR1*. 75 random frames were used for 1, while the whole basin (69 frames) was used for 2

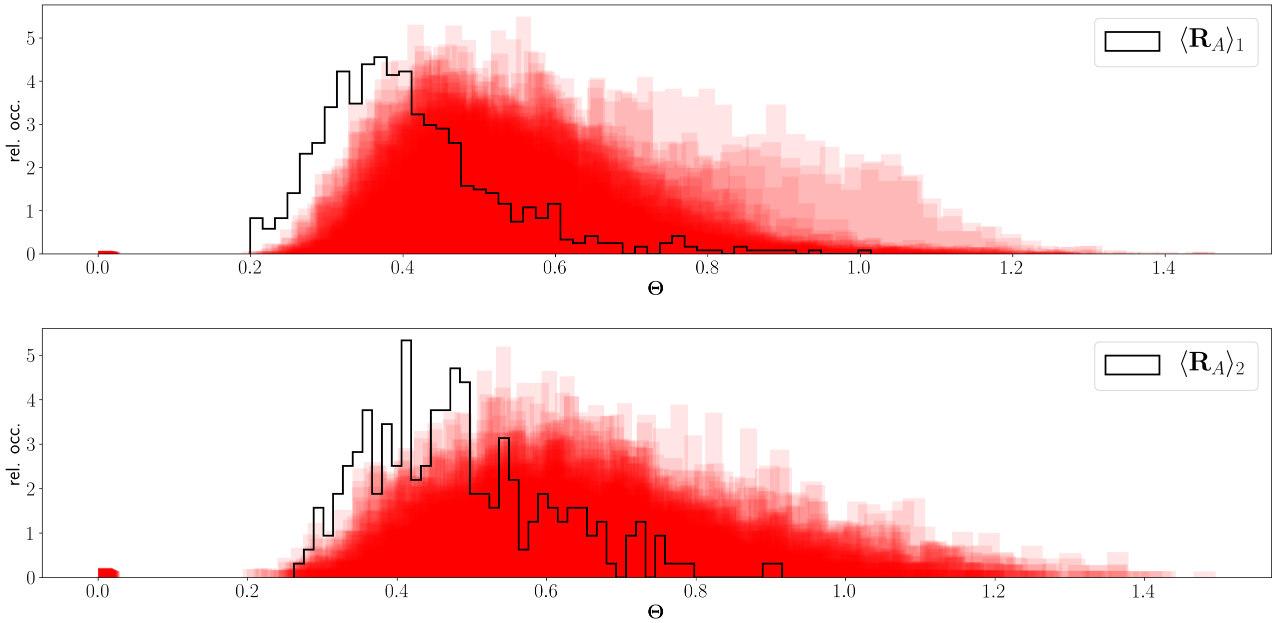

Figure S8: Distribution of Θ values, calculated for all atoms but methyl hydrogens, for each average structure (black line) and for 75 randomly selected frames (low transparency red bars) for *AT-JSR1*.

S2 Observables from ensemble and geometric averaging

Table S1: Ensemble averaging($\langle f \rangle$) vs geometric averaging for single geometries($\tilde{f}_k$) either from single basins or from direct averaging. Errors are defined as $\Delta f = \tilde{f}_k - \langle f \rangle_k$.

system	k	$\tilde{f}^{iso}$	$\langle f^{iso} \rangle$	$\tilde{f}^{emb}$	$\langle f^{emb} \rangle$	Δf^{iso}	Δf^{emb}	N_{frames}
AT-C1C2	1	2.504	2.413	2.629	2.543	0.091	0.086	497
AT-C1C2	2	2.497	2.414	2.620	2.551	0.083	0.069	219
AT-C1C2	3	2.471	2.413	2.624	2.559	0.059	0.065	31
AT-C1C2	$1 \cup 2 \cup 3$, [1]	1.515	2.413	1.781	2.546	-0.898	-0.764	750
AT-C1C2	$1 \cup 2 \cup 3$, [2]	2.519	2.413	2.642	2.546	0.105	0.097	750
9C-JSR1	1	1.762	1.681	1.842	1.768	0.080	0.074	691
9C-JSR1	2	1.764	1.680	1.853	1.774	0.084	0.079	165
9C-JSR1	3	1.745	1.651	1.803	1.727	0.094	0.077	99
9C-JSR1	$1 \cup 2 \cup 3$	1.772	1.678	1.850	1.765	0.094	0.085	1000
11C-JSR1	1	1.566	1.507	1.594	1.545	0.059	0.049	863
11C-JSR1	2	1.578	1.531	1.606	1.569	0.047	0.037	69
11C-JSR1	$1 \cup 2$	1.574	1.512	1.602	1.550	0.062	0.052	1000
AT-JSR1	1	1.887	1.800	1.909	1.826	0.087	0.083	741
AT-JSR1	2	1.997	1.901	2.020	1.930	0.096	0.090	244
AT-JSR1	$1 \cup 2$	1.929	1.827	1.952	1.854	0.101	0.098	1000

Table S2: Ensemble averaging($\langle f \rangle$) vs geometric averaging for the weighted average of basins($\langle \tilde{f} \rangle_{1,\ldots,K}$). Errors are defined as $\Delta f = \langle \tilde{f}_k \rangle_{1,\ldots,K} - \langle f \rangle_N$.

system	k	$\langle \tilde{f}^{iso} \rangle$	$\langle f^{iso} \rangle$	$\langle \tilde{f}^{emb} \rangle$	$\langle f^{emb} \rangle$	Δf^{iso}	Δf^{emb}
AT-C1C2	$1, 2, 3$	2.501	2.413	2.626	2.546	0.088	0.080
9C-JSR1	$1, 2, 3$	1.760	1.678	1.840	1.765	0.082	0.075
11C-JSR1	$1, 2$	1.567	1.512	1.595	1.550	0.056	0.045
AT-JSR1	$1, 2$	1.914	1.827	1.936	1.854	0.087	0.083

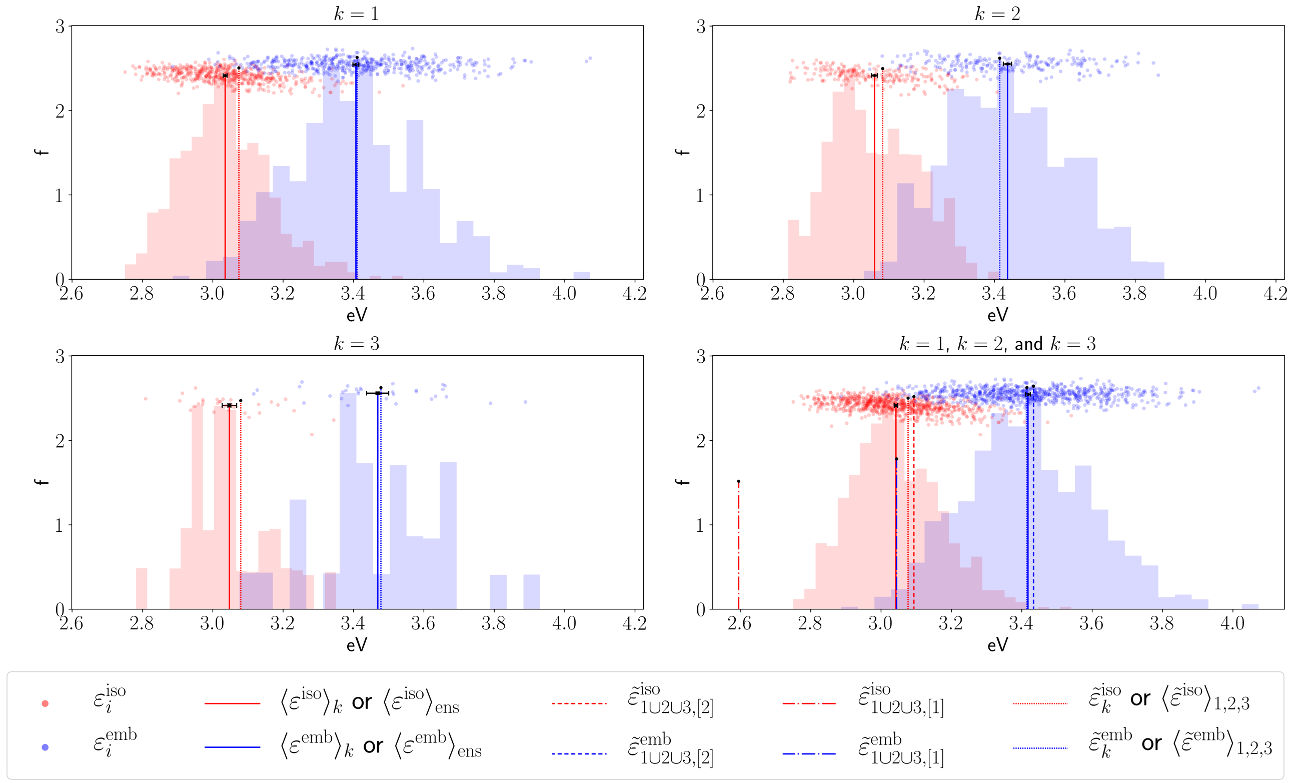

Figure S9: Ensemble and geometric, for all basins for *AT-C1C2*. Ensemble, geometric, and direct averaging for all basins(k). Legend refers to ε but applies also to f. The red and blue with low transparency represent the oscillator-weighted distribution of excitation energies for the iso and emb respectively. The distributions are normalised so that their maximum matches the oscillator strength of the ensemble averaged value. Both structures from direct geometric averaging are shown.

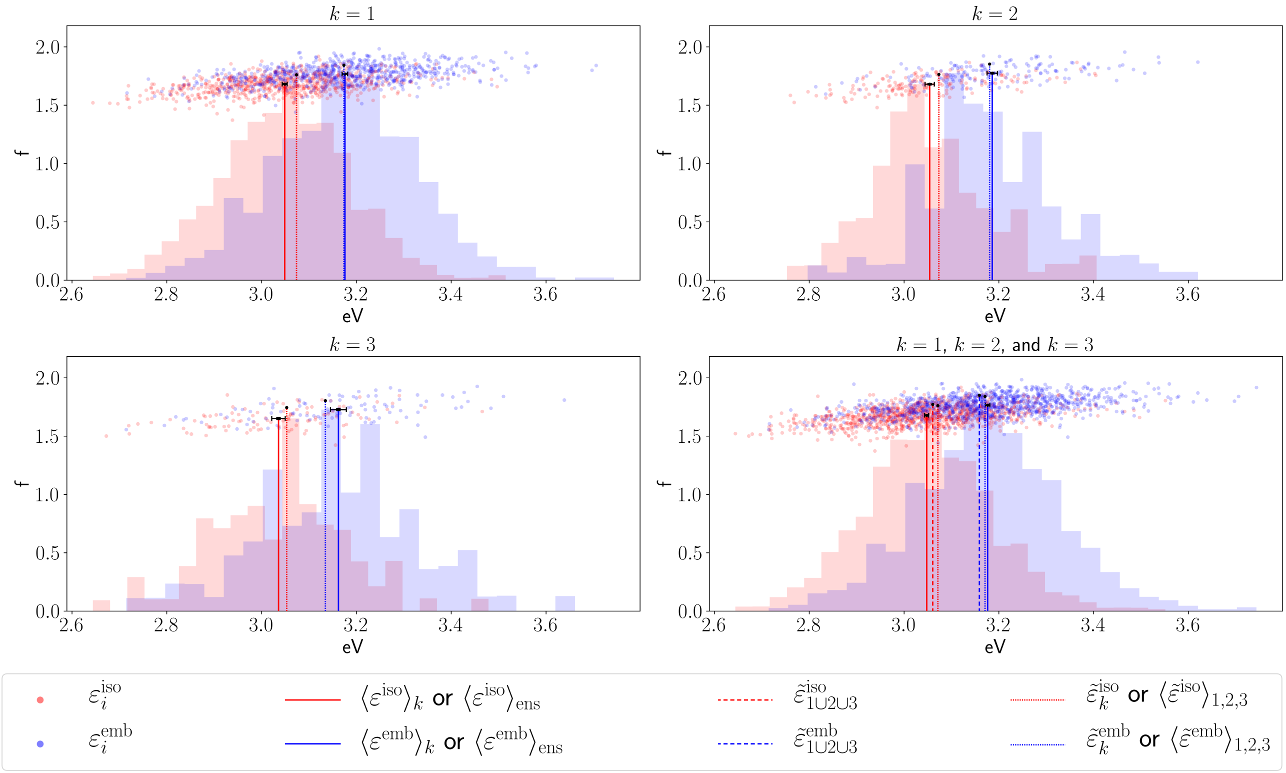

Figure S10: Ensemble and geometric, for all basins for *9C-JSR1*. Ensemble, geometric, and direct averaging for all basins(k). Legend refers to ε but applies also to f. The red and blue with low transparency represent the oscillator-weighted distribution of excitation energies for the iso and emb respectively. The distributions are normalised so that their maximum matches the oscillator strength of the ensemble averaged value.

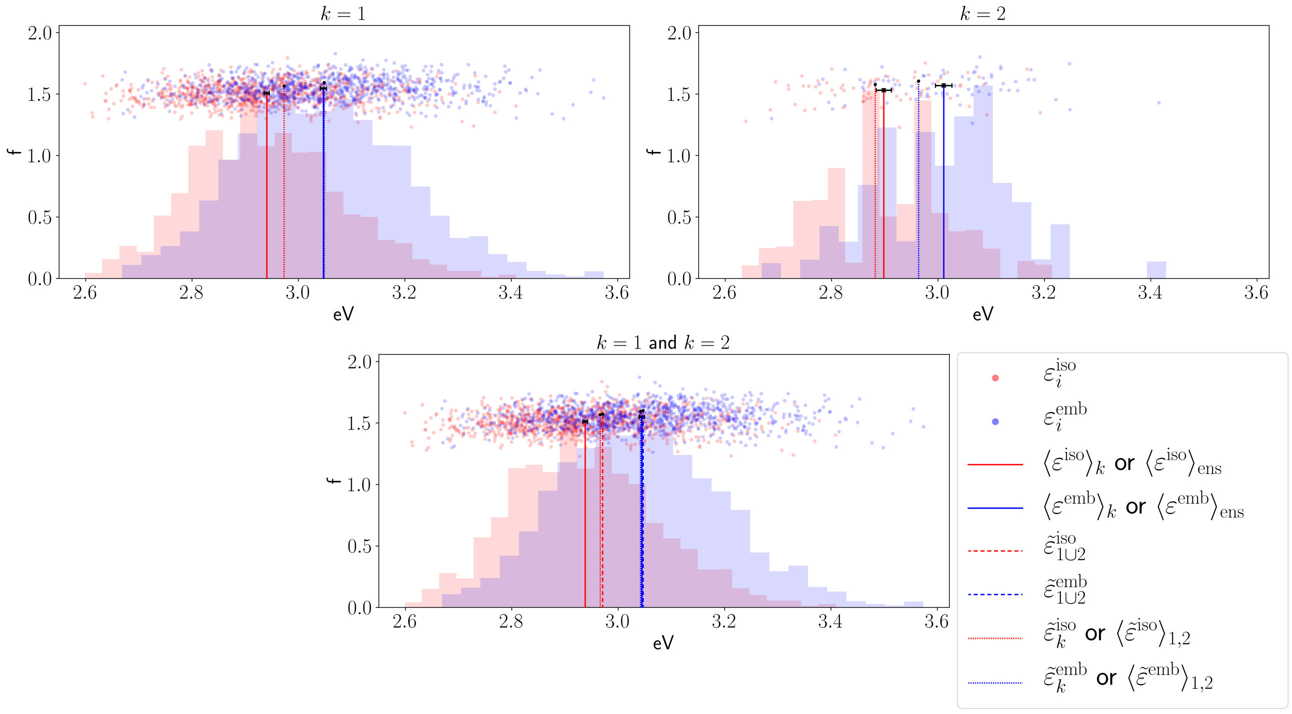

Figure S11: Ensemble and geometric, for all basins for *11C-JSR1*. Ensemble, geometric, and direct averaging for all basins(k). Legend refers to ε but applies also to f. The red and blue with low transparency represent the oscillator-weighted distribution of excitation energies for the iso and emb respectively. The distributions are normalised so that their maximum matches the oscillator strength of the ensemble averaged value.

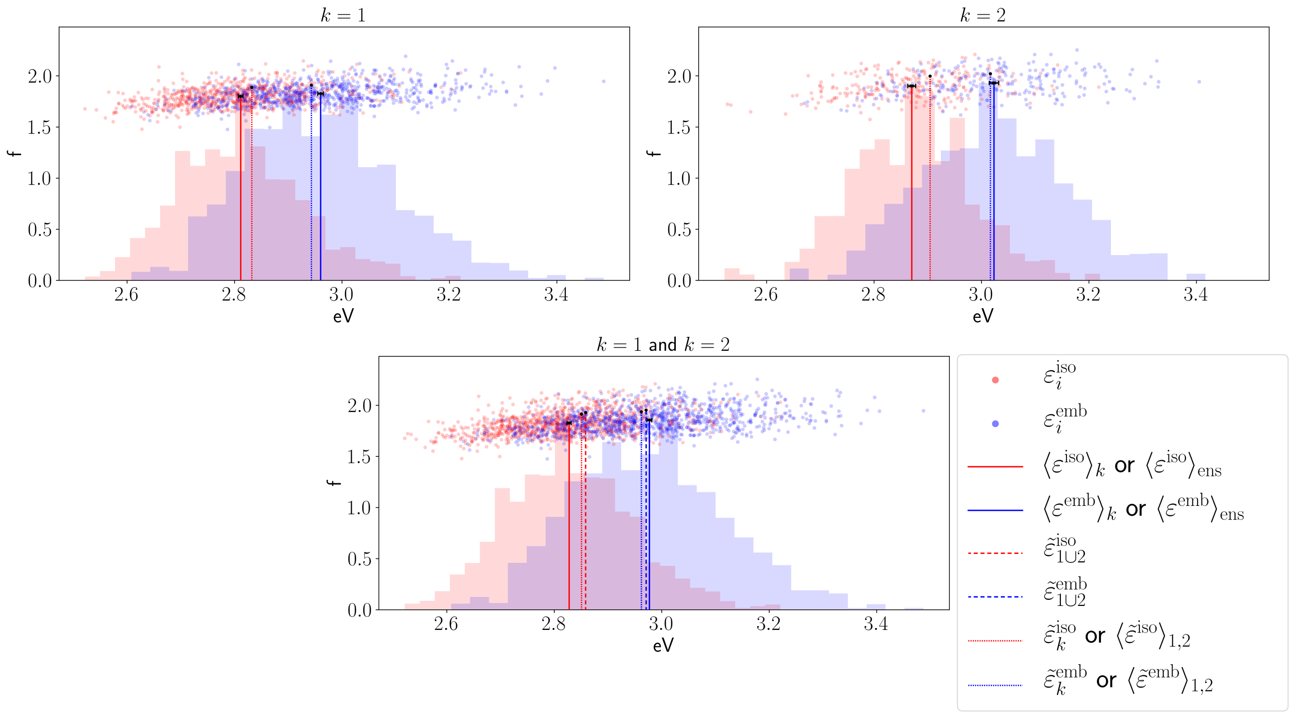

Figure S12: Ensemble and geometric, for all basins for *AT-JSR1*. Ensemble, geometric, and direct averaging for all basins(k). Legend refers to ε but applies also to f. The red and blue with low transparency represent the oscillator-weighted distribution of excitation energies for the iso and emb respectively. The distributions are normalised so that their maximum matches the oscillator strength of the ensemble averaged value.

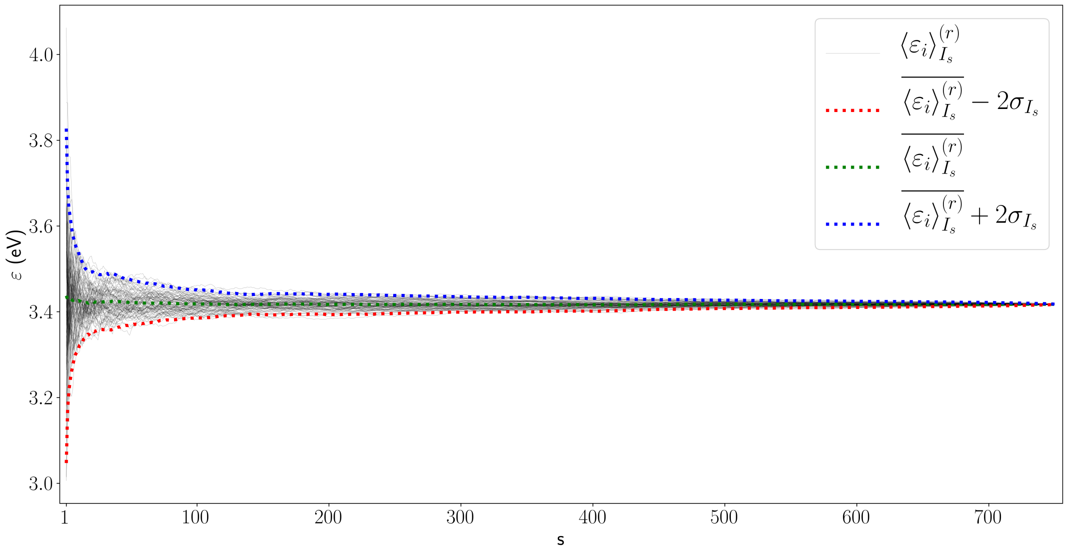

Figure S13: Evolution of the ensemble averaged excitation energy $\langle \varepsilon_i \rangle_{I_s}^{(r)}$ of embedded *AT-C1C2* for incrementally larger randomly selected subensembles (full black lines). 100 such lines are represented ($r = 1, \ldots, 100$). Their average $\overline{\langle \varepsilon_i \rangle_{I_s}^{(r)}}$ is shown as a dashed green line. The lower $(\overline{\langle \varepsilon_i \rangle_{I_s}^{(r)}} - 2\sigma_{I_s})$ and upper $(\overline{\langle \varepsilon_i \rangle_{I_s}^{(r)}} + 2\sigma_{I_s})$ limits of the confidence interval are shown as a red and blue dashed line respectively.

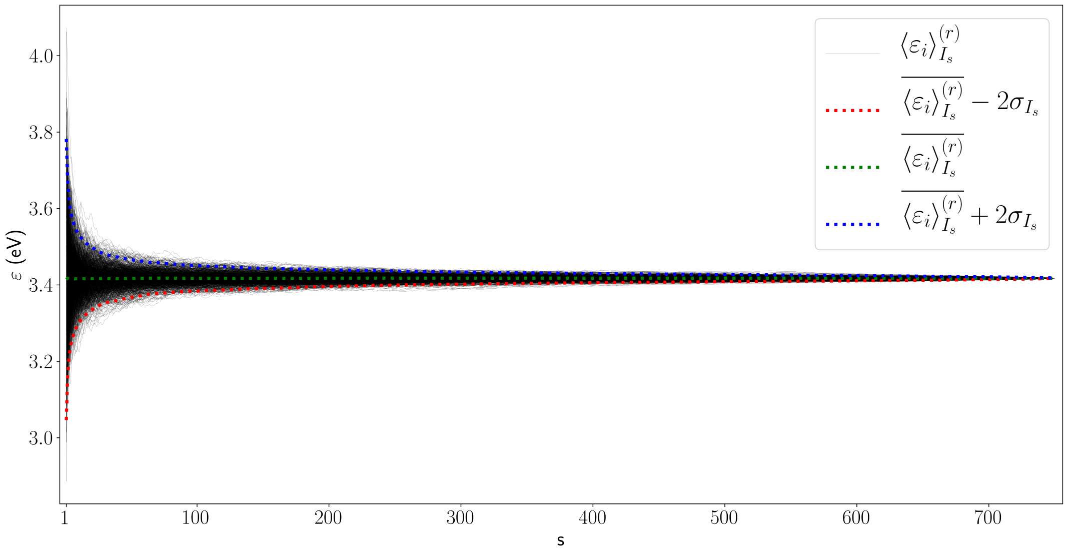

Figure S14: Analogous Figure to Fig. S13 but where 1000 different sets are shown ($r = 1, \ldots, 1000$).

S3 Bond Length Alternation

Bond Length Alternation (BLA) was calculated as the difference between the average single bond length and the average double bond length, where the single bonds are those between the five atom pairs 6–7, 8–9, 10–11, 12–13, and 14–15, and the double bonds are those between the five atom pairs 5–6, 7–8, 9–10, 11–12, and 13–14 (cf Fig. S15).

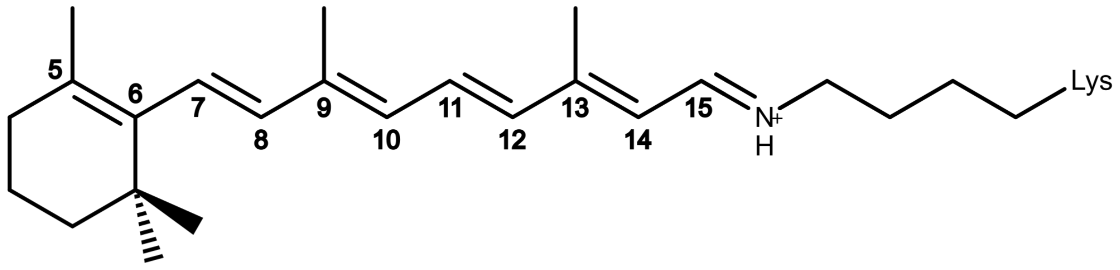

Figure S15: Two-dimensional representation of all-trans retinal protonated Schiff base.

The numerical values relating to the excitation energies in the various BLA-based bins are shown in Table S3.

Table S3: BLA value, ensemble and geometric averaging excitation energies, isolated and embedded. The average BLA value among the frames of a bin is numerically equal to the weighted average of the conformers' average structures within the bin.

bin	BLA	$\langle\tilde{\varepsilon}^{iso}\rangle_{1,2}$	$\langle\varepsilon^{iso}\rangle_{1,2}$	$\langle\tilde{\varepsilon}^{emb}\rangle_{1,2}$	$\langle\varepsilon^{emb}\rangle_{1,2}$
1	0.010	2.833	2.814	3.037	3.052
2	0.030	2.926	2.894	3.177	3.181
3	0.050	3.031	2.995	3.339	3.345
4	0.069	3.136	3.096	3.494	3.502
5	0.090	3.270	3.224	3.691	3.675
6	0.109	3.419	3.392	3.859	3.890
